# Autophagy-phytohormone crosstalk: a dual-regulation axis in plant development

**DOI:** 10.3389/fpls.2026.1781813

**Published:** 2026-02-26

**Authors:** Qing Pang, Min Tian, Tao Cao, Chunfa Chen, Weiming Hu, Fen Liu

**Affiliations:** 1 Jiangxi Provincial Key Laboratory of Plant Germplasm Resources Innovation and Genetic Improvement, Lushan Botanical Garden, Jiangxi Province and Chinese Academy of Sciences, Jiujiang, China; 2 Research Institute of Subtropical Forestry, Chinese Academy of Forestry, Hangzhou, China; 3 Jiangxi Wuyuan Tea Vocational College, Shangrao, China; 4 Jiangxi Academy of Forestry, Nanchang, China

**Keywords:** *ATG* genes, autophagy, nutrient deficiency, phytohormones, plant growth and development, stress response

## Abstract

Autophagy, a conserved catabolic process that degrades and recycles intracellular components, plays a pivotal role in maintaining plant growth, development, and stress tolerance. Emerging evidence indicates that autophagy is tightly interconnected with phytohormone signaling networks, which coordinate plant physiological processes through transcriptional and metabolic regulation. Recent studies have revealed a bidirectional regulatory relationship between these systems. Autophagy modulates phytohormone pathways by selectively degrading key signaling components, such as repressors in jasmonic acid signaling and regulators of abscisic acid responses, thereby fine-tuning hormonal outputs. Conversely, phytohormones including abscisic acid, ethylene, and salicylic acid can directly influence autophagy activity by controlling the expression of autophagy-related genes and thus the formation of autophagosomes. This dynamic crosstalk enables plants to integrate developmental programs with environmental cues. Here, we systematically summarize the most comprehensive and latest advances in our understanding of how autophagy and phytohormones coordinately regulate plant physiological processes. We also propose unresolved questions and future research directions to advance our knowledge of this essential regulatory network.

## 1 Introduction

Autophagy is an intracellular material degradation pathway that recycles cellular components during development or under stress conditions, which is highly conserved in eukaryotes. Plant autophagy can be categorized into microautophagy, macroautophagy, and mega-autophagy, with macroautophagy being the dominant form (Marshall and Vierstra, 2018), and it will be called autophagy hereafter. This process is closely associated with numerous core physiological processes in the plant life cycle, such as nutrient recycling (Shi et al., 2023; Yu et al., 2025), growth and development regulation (Cheng et al., 2022; Wang et al., 2022a, Wang et al., 2024b), stress resistance (Han et al., 2011; Zhang et al., 2021; Wang et al., 2022a; Cheng et al., 2025), and microbe interactions (Cheng et al., 2024).

Autophagy requires extensive membrane remodeling. A double-layered cup-shaped isolation membrane expands and engulfs cytoplasmic cargos. The isolation membrane closes and becomes an autophagosome with double-membrane, the outer membrane fuses with the tonoplast to release its internal cargo for degradation in the vacuole via vacuolar hydrolases (Feng et al., 2014).From a molecular perspective, the autophagy process relies on approximately 40 core autophagy-related (*ATG*) genes, and many ATG proteins are encoded by gene families in plants (Zhou et al., 2022). These ATG proteins make up four complexes essential for the autophagy process: (1) Autophagy begins with the phosphorylation of two key proteins, ATG1 and ATG13, and then they bind with two additional subunits ATG11 and ATG101 to form a complex, which together stimulate several downstream autophagy steps (Suttangkakul et al., 2011; Li et al., 2014); (2) ATG9, along with its cycling factors ATG2 and ATG18, promotes the transport of lipids to the expanding phagophore (Mann et al., 2023); (3) The decoration of the phagophore with phosphatidylinositol-3-phosphate (PI3P) by the class-III phosphatidylinositol-3-kinase (PI3K) complex containing VACUOLAR PROTEIN SORTING 34 (VPS34), VPS15, ATG6, and VPS38 or ATG14 (Liu et al., 2018, Liu et al., 2020); (4) The ATG5/ATG12/ATG16 E3 ligase complex functions in conjugation of ATG8 with phosphatidylethanolamine (PE), which is crucial for anchoring ATG8 to the phagophore (Zhang et al., 2022).

Autophagy occurs extensively in response to various environmental stresses or during certain stages of plant growth and development. To ensure rapid adaptation to the ever-changing environment, the intensity of autophagy is typically precisely regulated to maintain cellular homeostasis in a timely and appropriate manner. The regulation of autophagic activity largely depends on target of rapamycin (TOR) protein kinase complexes and SNF-related kinase 1 (SnRK1) (Feng et al., 2025). TOR acts as a negative regulator of autophagy in plants. Under nutrient-rich conditions, TOR is active and inhibits autophagy by phosphorylating ATG13 to prevent its binding to ATG1. However, under nutrient deprivation, TOR is inactivated, leading to the dephosphorylation of ATG13 and its subsequent binding to ATG1, which initiates autophagy (Mugume et al., 2020). SnRK1 is a positive regulator of plant autophagy, which activates autophagy through two mechanisms: (1) Inhibiting TOR activity (Soto-Burgos and Bassham, 2017) and (2) Directly phosphorylating ATG1 (Chen et al., 2017).

Importantly, accumulating evidence indicates that autophagy does not function as an isolated degradation pathway but is tightly integrated with phytohormone signaling networks. Multiple phytohormones have been reported to influence autophagy activity, often through central metabolic regulators such as TOR and SnRK1 or through transcriptional regulation of *ATG* genes. Conversely, autophagy can modulate phytohormone homeostasis and signaling outputs by selectively degrading hormone biosynthetic enzymes, receptors, or key signaling components. Through these bidirectional interactions, autophagy and phytohormone pathways together coordinate plant growth, development, and stress adaptation at the cellular and organismal levels. However, despite increasing evidence for such crosstalk (Gou et al., 2019; Li et al., 2020; Liao and Bassham, 2020; Liao et al., 2022), the underlying molecular mechanisms and their context dependency remain incompletely understood. Given this emerging evidence that autophagy is closely intertwined with phytohormone signaling, it is particularly important to examine the molecular mechanisms underlying autophagy–phytohormone interactions.

**Figure 1:**
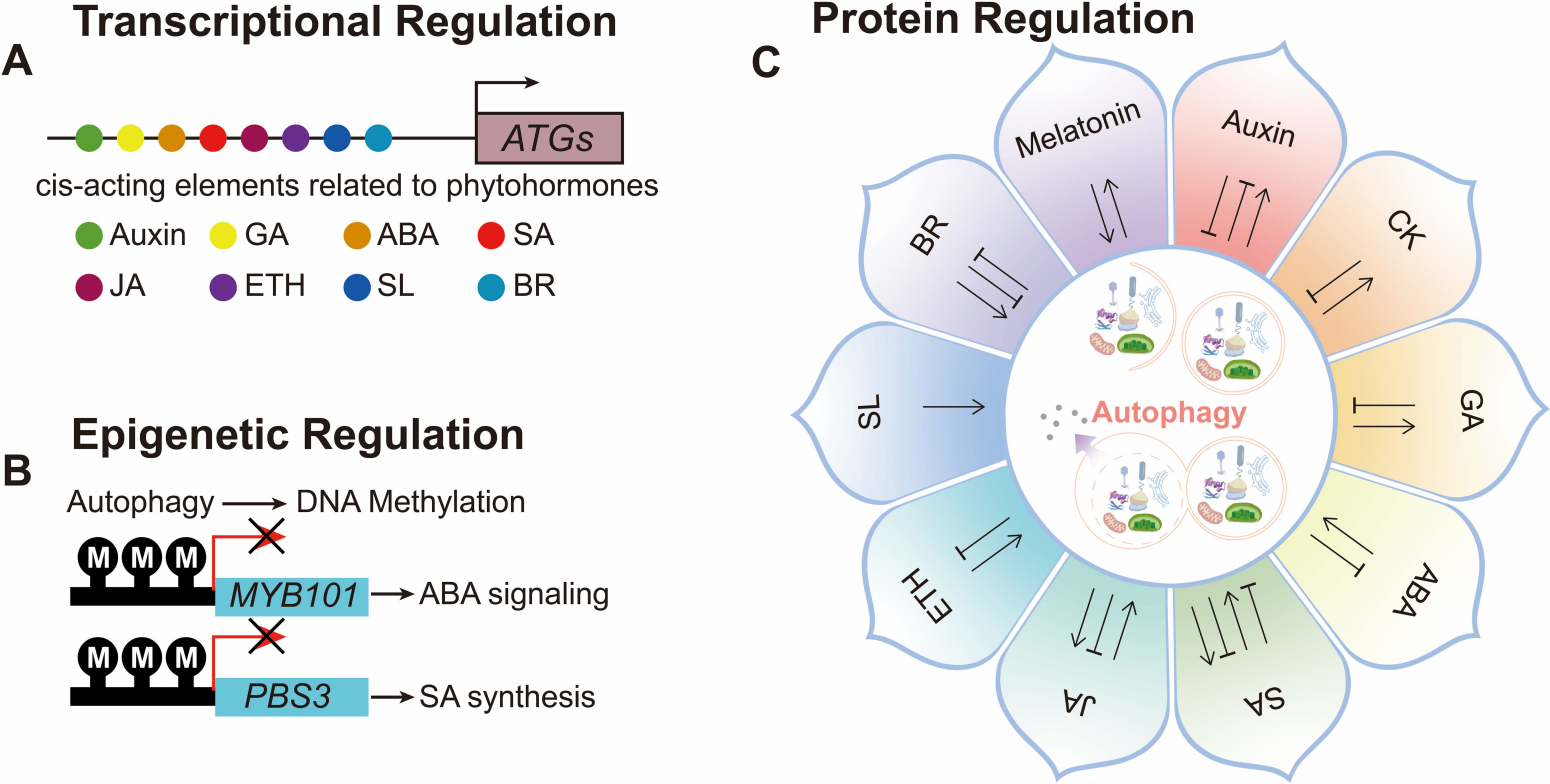
Schematic diagram of multilevel bidirectional regulation between phytohormones and autophagy. **(A)** Bioinformatics analysis predicted that autophagy-related genes (*ATGs*) contain *cis*-acting elements responsive to various phytohormones, including auxin, gibberellin (GA), abscisic acid (ABA), salicylic acid (SA), jasmonic acid (JA), ethylene (ETH), strigolactones (SL), and brassinosteroids (BR). **(B)** At the epigenetic level, autophagy can regulate key gene expression through DNA methylation, such as the regulation of *MYB101* in ABA signaling and *PBS3* in SA synthesis. **(C)** At the protein level, various phytohormones exhibit bidirectional regulatory relationships with autophagy.

In this review, we summarize recent advances in understanding the molecular basis of autophagy–phytohormone crosstalk. We focus on how different phytohormones influence autophagy activity and how autophagy modulates hormone biosynthesis, signaling, and downstream physiological processes, particularly in the context of environmental stress responses and key developmental stages. By integrating these findings, we aim to provide an updated framework for understanding how autophagy functions as an important regulatory hub within plant hormone networks (**Figure 1**; **Supplementary Tables S1-S3**).

## 2 Auxin

### 2.1 Regulation of autophagy by auxin

Auxin plays a significant role in regulating autophagy-mediated physiological processes that enable plants to cope with various abiotic stresses. When plants encounter abiotic stresses such as sucrose starvation, nitrogen starvation, salt stress, or osmotic stress, exogenous auxin application significantly inhibits autophagy activation. Subsequent experiments confirmed that auxin acts upstream of the TOR pathway in the autophagy process (Beltrán-Peña et al., 2002; Turck et al., 2004; Schepetilnikov et al., 2013; Li et al., 2017; Pu et al., 2017; Schepetilnikov et al., 2017; Kazibwe et al., 2020). These studies collectively reveal that auxin may regulate autophagy through the TOR signaling pathway, thereby assisting plants in coping with various stresses.

Furthermore, studies at the genetic level have provided additional insights into the relationship between auxin and autophagy. Three auxin-related elements are present in the promoters of wheat *ATGs* (Yue et al., 2018), 17 auxin-responsive *cis*-acting elements are found in the promoters of rice *ATGs* (Xie et al., 2024). And the promoter regions of *Arabidopsis ATG8a* and *ATG8h* contain binding sites for the auxin response factor (ARF) family (Wang et al., 2020). These findings strongly suggest that the auxin signaling pathway may regulate the expression of *ATGs* in response to environmental conditions, dynamically adjusting autophagy levels to ensure normal plant growth and development. However, the specific molecular mechanisms underlying this process remain to be further elucidated.

**Figure 2:**
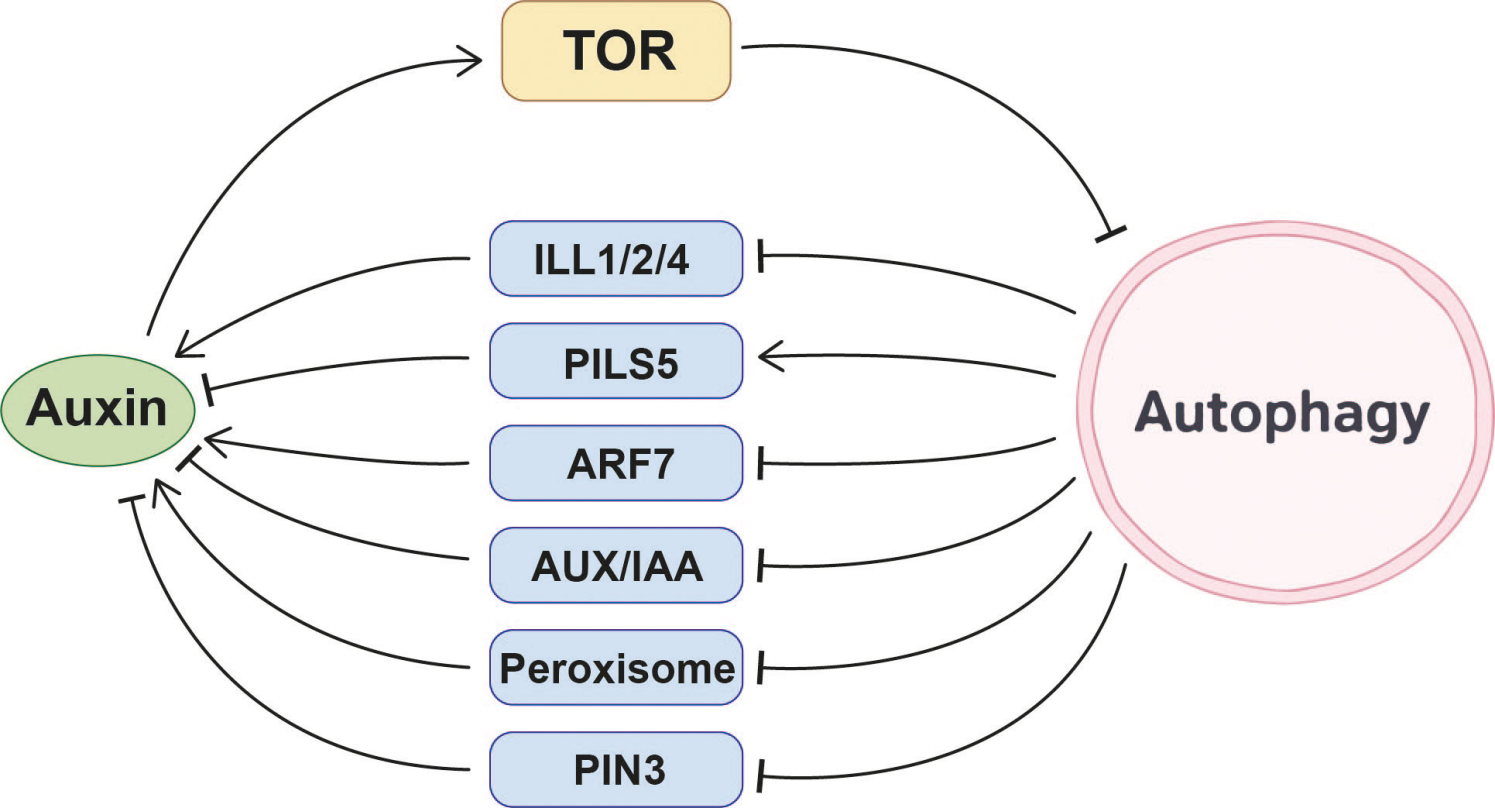
Molecular interplay mechanisms between auxin and autophagy.

In summary, current evidence consistently supports a model in which auxin generally suppresses autophagy, largely through activation of the TOR pathway, while also potentially regulating *ATG*s transcription via auxin-responsive elements. This positions auxin as an upstream hormonal signal that integrates growth status with autophagy activity under changing environmental conditions (**Figure 2**).

### 2.2 Regulation of auxin by autophagy

Autophagy plays a crucial role in regulating root development. Specifically, it participates in glucose-mediated modulation of root meristem activity by maintaining cellular homeostasis of auxin in *Arabidopsis* (Huang et al., 2019). Under sucrose excess stress, autophagy regulates root growth through a unique mechanism: autophagy enhances root sensitivity to sucrose excess, manifesting as growth inhibition. In contrast, autophagy-deficient *atg* mutants alleviate this inhibitory effect by accumulating more auxin-promoting proteins and reducing auxin transporters. Specifically, under sucrose excess treatment, increased levels of three IAA-amino acid hydrolases of the ILR1-like family proteins, ILL1, 2, and 4, were observed in *atg5–1* mutant roots. These proteins can lead to the accumulation of active auxin. Meanwhile, the auxin transporter PIN-LIKES 5 (PILS5) is downregulated, retaining auxin within cells and further elevating the level of active auxin in the cytoplasm. Consequently, the primary root growth of wild-type *Arabidopsis* is significantly inhibited under sucrose excess, whereas the roots of *atg* mutants show less severe inhibition and greater root length (Laloum et al., 2022). It should be noted that the above conclusions are based on protein abundance changes, and it remains unclear whether these auxin-related proteins are directly degraded through autophagy or are indirectly affected through other regulatory pathways. During lateral root formation, autophagy directly binds to and regulates the periodic fluctuation and quantitative balance of the auxin response factor ARF7 through NBR1-mediated selective autophagy, thereby influencing the regulation of lateral root formation by the auxin signaling pathway. ARF7 accumulates in *atg* mutants, disrupting the expression of its downstream genes and impairing the normal program of lateral root formation (Ebstrup et al., 2024).

In response to Pi starvation, Pi deficiency triggers *Arabidopsis* Receptor Kinase 2 (AtARK2) phosphorylation-mediated activation of PUB9. Subsequently activated PUB9, acting as an E3 ubiquitin ligase, marks AUX/IAA proteins or other auxin-accumulating inhibitors for autophagy degradation. This consequently leads to the accumulation of auxin in lateral root primordia, inducing an increase in lateral roots to cope with Pi starvation (Deb et al., 2014; Sankaranarayanan and Samuel, 2015).

In the regulation of leaf senescence, the aging process of maize leaves is modulated by the interaction between autophagy and auxin. Using two maize lines, Si-287 (early senescence) and Si-144 (stay-green), as research subjects, population genetics and transcriptomics analyses revealed that the *ZmATG18b* exhibits higher expression in the early senescence line Si-287. This gene accelerates autophagy-mediated degradation of cellular components, thereby promoting leaf senescence. The auxin synthesis-related gene *ZmGH3.8* exhibits higher expression in the stay-green line Si-144, delaying senescence by regulating auxin levels. The dynamic balance of expression levels of these two gene plays a crucial role in the process of maize leaf senescence (Feng et al., 2021).

In regulating plant heat tolerance, autophagy participates in the response to high temperatures by influencing auxin synthesis and distribution. In the heat-sensitive *Arabidopsis fes1a* mutant, autophagy indirectly inhibits auxin synthesis by degrading peroxisomes, thereby reducing the plant’s heat tolerance. The *fes1a* mutant exhibits reduced heat tolerance due to the absence of the chaperone protein FES1A. However, heat tolerance unexpectedly recovers when autophagy is blocked. One reason is that blocking autophagy leads to massive accumulation of peroxisomes in the *atg7 fes1a* double mutant. Peroxisomes promote auxin synthesis, and the accumulation of auxin in plants enhances heat tolerance, mitigating the heat-sensitive phenotype (Li et al., 2025). Additionally, autophagy activated in *Arabidopsis* under high-temperature conditions enhances heat dissipation by promoting hypocotyl elongation through regulating auxin distribution. High temperatures activate the transcription factor phytochrome-interacting factor 4 (PIF4), which initiates expression of the key actin cytoskeleton assembly gene ArpC5. Activated ArpC5 promotes actin cable formation, providing structural support for autophagosome assembly. Simultaneously, ArpC5 binds to the ATG8, facilitating autophagosome movement along actin filaments to ensure efficient autophagy progression. Active autophagy degrades the auxin transporter PIN3, which helps regulate auxin distribution within cells. This concentrates auxin in the elongation zone of the hypocotyl, enabling plants to enhance heat dissipation by elongating the hypocotyl under high-temperature conditions (Pan et al., 2025).

Collectively, these studies demonstrate that autophagy modulates auxin homeostasis at multiple levels, including auxin biosynthesis, transport, signaling components, and auxin-responsive developmental programs. Through selective degradation of key regulators, autophagy fine-tunes auxin-dependent root architecture, stress adaptation, and organ development. However, whether certain proteins in the auxin signaling pathway serve as direct substrates of autophagy remains, to some extent, unclear or experimentally unverified, and this represents an important direction for future research (**Figure 2**).

## 3 Cytokinin

### 3.1 Regulation of autophagy by CK

Emerging evidence indicates that CK exerts an inhibitory effect on autophagy. A post-harvest physiological study of plants investigated the regulation of autophagy by CK. 6-benzylaminopurine (6-BA) is a synthetic CK analog. Treatment with 6-BA upregulates the expression of genes associated with sucrose synthesis. Sufficient sugar levels inhibit the degradation of Rubisco by suppressing the expression of *ATGs*, thereby alleviating chlorophyll degradation in *Brassica rapa* Subsp. *Chinensis* and delaying yellowing during storage (Song et al., 2025).

Although direct mechanistic evidence remains limited, current findings suggest that CK tends to repress autophagy, possibly through its role in maintaining nutrient and carbon status. This highlights a potential link between CK-mediated growth signals and autophagy suppression that warrants further investigation.

### 3.2 Regulation of CK by autophagy

The research by Silvia Slavikova et al. first proposed the association between autophagy and CK signaling. They found that the GFP-AtAtg8f-HA fusion protein affects cytokinin-mediated regulation of root architecture and root–shoot communication (Slavikova et al., 2008). Exogenous zeatin treatment significantly reduced the number of autophagosome-like structures in root epidermal cell vacuoles, but induced the formation of GFP-AtAtg8f-containing structures near deeper vascular tissues. These structures are hypothesized to sequester proteins involved in cytokinin transport or signaling, thereby abrogating root–shoot communication (Slavikova et al., 2008).

Research by Atiako Kwame Acheampong et al. indicates that autophagy can fine-tune cellular sensitivity to CK by regulating the levels of type-A ARR proteins (Acheampong et al., 2020). Specifically, autophagy relieves its inhibition on CK signaling by degrading type-A ARR proteins. In rice, related studies similarly revealed an association between autophagy and CK metabolism: the Tz (trans-zeatin) content in the anthers of the autophagy-deficient mutant *Osatg7–1* was reduced, suggesting that autophagy may possess a conserved function in maintaining CK homeostasis in plants (Kurusu et al., 2017).

Transcriptome-level analysis provides further evidence for the association between autophagy and CK: genes upregulated in the autophagy-deficient mutant *atg5* are also upregulated in the CK receptor triple mutant *ahk2 ahk3 ahk4*, while they are downregulated in the 6-BA-treated *arr1 arr10 arr12* triple mutant (B-type CK response regulator mutant). This indicates that autophagy-deficient mutants may exhibit defects in cytokinin perception and response, suggesting that autophagy may be involved in CK signaling pathways (Masclaux-Daubresse et al., 2014). This data further supports the research of Atiako Kwame Acheampong et al (Acheampong et al., 2020). and Takamitsu Kurusu et al (Kurusu et al., 2017).

**Figure 3:**
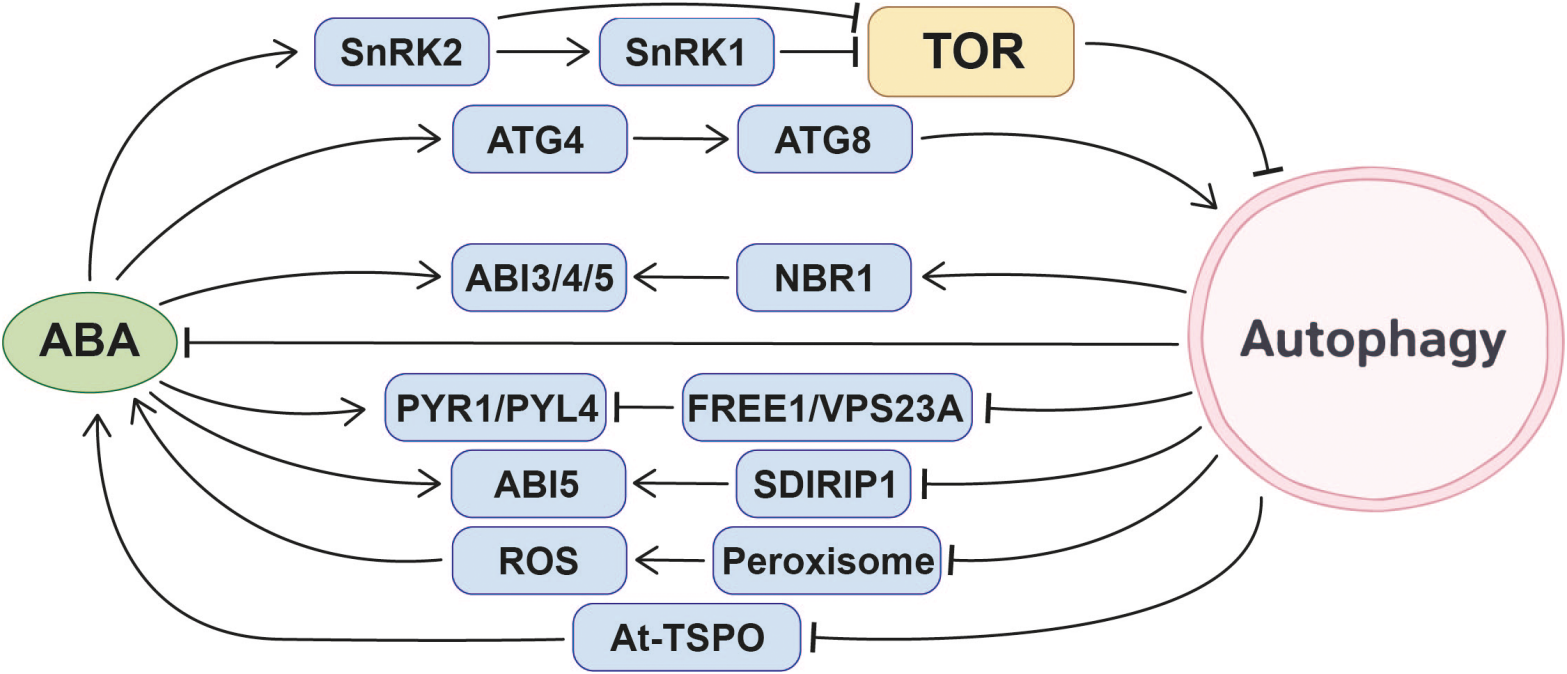
Molecular interplay mechanisms between abscisic acid (ABA) and autophagy.

Together, these findings indicate that autophagy contributes to the fine-tuning of CK signaling and homeostasis, possibly by modulating the abundance of key CK response regulators and influencing CK metabolism (**Figure 3**).

## 4 Gibberellin

### 4.1 Regulation of autophagy by GA

GA participates in the regulation of plant autophagy through multiple pathways, and the mechanism has been partially elucidated in different plant species. Research on the storage and freshness preservation of *Brassica rapa* Subsp. *Chinensis* has revealed that GA treatment significantly upregulates the expression of genes associated with sucrose synthesis. This regulatory effect promotes the accumulation of sufficient sugars within the plant. The sugar signal inhibits the degradation of Rubisco by suppressing the expression of autophagy-related genes, thereby mitigating chlorophyll degradation in *Brassica rapa* Subsp. *Chinensis*, thereby reducing yellowing during storage (Song et al., 2025). Furthermore, analysis of *cis*-acting elements in gene promoter regions provides molecular evidence for the association between GA and autophagy. Three GA-related elements were identified in the promoters of wheat *ATGs* (Yue et al., 2018). Twenty GA-responsive *cis*-acting elements are present in the promoters of rice *ATGs* (Xie et al., 2024). These findings suggest that the GA signaling pathway may dynamically regulate plant autophagy in response to environmental conditions to ensure normal plant growth and development.

Collectively, current studies suggest that GA may suppress autophagy, partly through sugar-related signaling, while promoter analyses imply a broader transcriptional connection between GA signaling and autophagy regulation. However, the direct signaling links between GA and the core autophagy machinery remain largely unresolved.

### 4.2 Regulation of GA by autophagy

Autophagy plays a crucial role in the sexual reproductive development of rice. Research indicates that the autophagy-deficient rice mutants *Osatg7–1* and *Osatg9* exhibit sporophyte male sterility under normal growth conditions, with significantly restricted anther dehiscence. Further phytohormone content assays revealed that endogenous levels of active form GAs in the anthers of the *Osatg7–1* were significantly lower than those in the wild-type, while exogenous application of GA4 can partially restore pollen maturation in the mutant. This result confirms that autophagy can influence pollen maturation by regulating the metabolism and endogenous levels of GAs in rice anthers (Kurusu et al., 2017).

Given the antagonistic regulatory relationship between GA and abscisic acid (ABA) in numerous developmental processes and responses to biotic and abiotic stresses, investigating the potential mechanisms underlying GA-ABA antagonism in autophagy regulation holds significant theoretical merit. Previous studies have revealed that SnRK2 in rice participates in the antagonistic regulation process between ABA and GA. ABA activates SnRK2, and the activated SnRK2 inhibits GA signal transduction (Lin et al., 2015), while SnRK2 is a key participant in stress-induced autophagy (Wang et al., 2018; Signorelli et al., 2019). Linking autophagy to the GA-ABA antagonism via SnRK2 kinase represents one of the key entry points for exploring the connection between autophagy and the GA signaling pathway. However, to date, the specific molecular mechanisms underlying this pathway remain unclear and require further investigation in subsequent studies.

**Figure 4:**
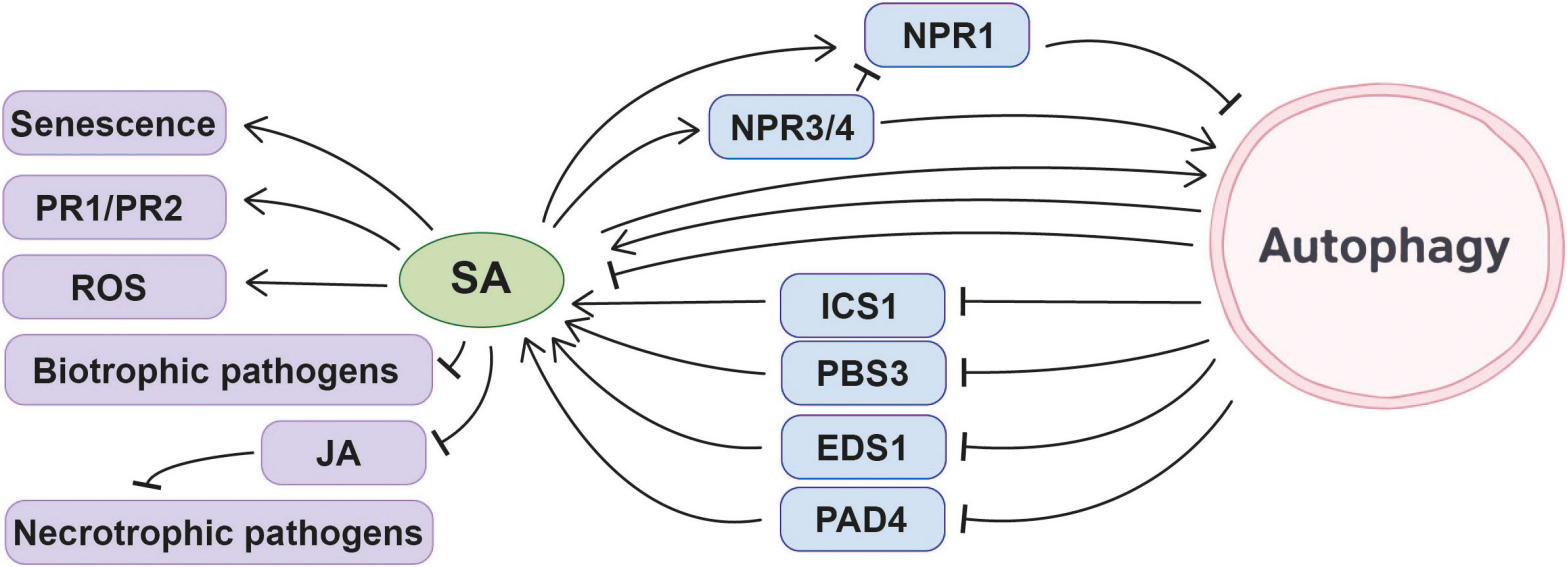
Molecular interplay mechanisms between salicylic acid (SA) and autophagy.

These observations demonstrate that autophagy is required for maintaining proper GA homeostasis during reproductive development, particularly in rice anthers. This places autophagy as a potential modulator of GA-dependent developmental transitions (**Figure 4**).

## 5 ABA

### 5.1 Regulation of autophagy by ABA

Among phytohormones, ABA has a more extensively studied and mechanistically detailed relationship with autophagy. Multiple studies across different species provide strong evidence for ABA’s positive regulation of autophagy. Research focusing on the banana *MaATG8f* gene reveals that ABA treatment induces the formation of GFP-MaATG8f marked autophagosomes in transgenic plants, demonstrating that ABA can induce autophagy in bananas (Li et al., 2019). In rapeseed, the expression levels of *BnATGs* transcripts increase in response to ABA treatment (Eshkiki et al., 2020). After spraying strawberries with exogenous ABA, the protein content of FvRD21 significantly increased. The FvRD21 protein can bind to the P6 protein of the strawberry vein banding virus (SVBV), thereby degrading P6 through the autophagy pathway and inhibiting SVBV infection (Yang et al., 2025). In *Arabidopsis* seedlings, expressing the GFP-ATG8e fusion protein, treatment with 50 μM ABA for 3 or 6 hours resulted in significantly elevated free GFP protein levels compared to the control conditions. This increase was further corroborated by the significantly higher quantified GFP/GFP-ATG8 ratio, collectively indicating that ABA effectively induces autophagic flux (Laureano-Marín et al., 2020).

At least three mechanisms by which ABA induces autophagy have been identified so far. Under normal conditions, intracellular H_2_S maintains high sulfidation modification of ATG4a, inhibiting its activity and keeping autophagy at low levels. while ABA reduces sulfidation modification of ATG4a, activating it to cleave ATG8, thereby promoting ATG8 lipidation and advancing autophagosome formation, which enhances plant stress resistance (Laureano-Marín et al., 2020). When plants encounter drought stress, ABA activates SnRK2. Activated SnRK2 phosphorylates RaptorB, a component of the TOR complex, leading to dissociation of the TOR complex and inhibition of TOR activity (Wang et al., 2018).Simultaneously, SnRK1 is a downstream component of SnRK2. In the presence of ABA, SnRK2 activates SnRK1 signaling (Belda-Palazón et al., 2020). Activation of SnRK1 also inhibits TOR activity, a negative regulator of autophagy, leading to the induction of autophagy (Salem et al., 2018).

Additionally, ABA may regulate autophagy activity by modulating the expression of *ATGs*. Three ABA-responsive elements are present in the promoters of wheat *ATGs* (Yue et al., 2018). Thirty-four ABA-responsive *cis*-acting elements are present in the promoters of rice *ATGs* (Xie et al., 2024) These studies collectively suggest that the ABA signaling pathway may regulate autophagy according to environmental conditions to ensure normal plant growth.

In summary, ABA is a positive regulator of autophagy, acting through multiple mechanisms including TOR inhibition, SnRK-mediated signaling, ATG4 activation, and potential transcriptional control of *ATGs*. This positions ABA as one of the central phytohormonal activators of autophagy during abiotic stress responses (**Figure 3**).

### 5.2 Regulation of ABA by autophagy

Autophagy plays a multidimensional regulatory role in ABA signaling and physiological responses in plants. Its mode of action exhibits significant variations across species, genetic backgrounds, physiological states, and environmental conditions, specifically manifesting as induction or suppression of ABA responses, or participation in signaling balance through intricate mechanisms.

In certain experimental systems, the activation of autophagy can induce an ABA response. The endogenous ABA level in the leaves of *Arabidopsis* overexpressing banana ATG8f was significantly higher than that in WT leaves, and these plants exhibited enhanced drought tolerance (Li et al., 2019). Additionally, transcriptomic data revealed that ABA downstream response genes were upregulated in NBR1 overexpression line in *Arabidopsis*, and the NBR1 overexpression line exhibited ABA-sensitive symptoms such as delayed seed germination and enhanced stomatal closure. Further experiments demonstrate that NBR1 can interact with key ABA signaling transcription factors ABI3, ABI4, and ABI5, balancing ABA signaling by degrading or regulating the activity of these proteins. However, additional evidence is required to confirm whether autophagy directly participates in NBR1-mediated ABA signaling regulation (Tarnowski et al., 2020).

In other physiological contexts, autophagy exhibits an inhibitory effect on ABA signaling and synthesis. For example, in apple plants overexpressing *MdATG10*, the expression of genes involved in the ABA pathway is reduced, and ABA synthesis is also decreased. This regulation helps maintain normal stomatal opening, balancing water loss with the gas exchange required for photosynthesis, ultimately making the plants more drought-tolerant than the wild type (Xiang et al., 2024). During tomato fruit ripening, autophagy also suppresses endogenous ABA levels. Research has found that tomato fruits from autophagy mutants exhibit elevated ABA levels, suggesting autophagy may suppress ABA accumulation during fruit ripening by degrading related substances or regulating synthetic pathways (Guo et al., 2025a).

Autophagy participates in ABA signal regulation through multiple pathways. ESCRT components FYVE DOMAIN PROTEIN REQUIRED FOR ENDOSOMAL SORTING1 (FREE1) and VACUOLAR PROTEIN SORTING23A (VPS23A) dynamically regulate the ABA signaling pathway based on endogenous ABA levels. In the absence of ABA, FREE1 and VPS23A interact with ubiquitinated ABA receptors PYR1 and PYL4, leading to their degradation via the multi-vesicle body (MVB)-mediated vacuolar sorting pathway, thereby suppressing ABA responses (Belda-Palazon et al., 2016; Yu et al., 2016). When ABA accumulates within plants, it triggers the degradation of FREE1 and VPS23A via autophagy and proteasome, thereby releasing the degradation inhibition on ABA receptors and activating downstream signaling (Xia et al., 2020). In addition, SDIRIP1 (SDIR1-INTERACTING PROTEIN1) participates in ABA signaling regulation by selectively modulating the expression of ABI5 (ABA-INSENSITIVE5). SDIRIP1 itself may be degraded via the autophagy pathway, though the detailed degradation mechanism remains to be further investigated (Zhang et al., 2007, Zhang et al., 2015). In guard cells, the accumulation of damaged or aged peroxisomes leads to the excessive accumulation of ROS (reactive oxygen species), a key second messenger in ABA signaling, thereby triggering stomatal closure and affecting plant respiration. Under normal physiological conditions, autophagy maintains low ROS levels by clearing damaged or aged peroxisomes, thereby ensuring proper stomatal opening (Yamauchi et al., 2019).

Autophagy has a potential role in ABA storage and homeostasis. ABA is primarily stored in the form of ABA-GE (abscisic acid glucosyl ester) within vacuoles and the ER (endoplasmic reticulum) via several low-affinity pathways (Burla et al., 2013). AtBG1, as a β-glucosidase releasing active ABA, is primarily localized to the ER and possesses a conserved ER retention signal (Lee et al., 2006). Ivan Kulich et al. proposed a hypothesis that both ABA-GE and AtBG1 may enter the vacuole(and also, the apoplast) via autophagy-associated ER-to-vacuole transport pathways (Kulich and Žárský, 2014).

Autophagy also plays a role in the degradation of ABA-related proteins. When subjected to abiotic stresses, such as drought and high salinity, plants accumulate ABA, which induces the expression of certain related proteins. However, these proteins must be efficiently degraded after their transient existence, and autophagy plays a crucial role in this process. A compelling example demonstrates that selective autophagy can specifically degrade ABA-induced proteins such as At-TSPO. The degradation of At-TSPO via the autophagic pathway requires heme binding, though the detailed molecular mechanism remains to be elucidated (Vanhee and Batoko, 2011; Vanhee et al., 2011; Jurkiewicz and Batoko, 2018). Additionally, the ABA-induced PIP2;7-TSPO complex can also be degraded via the autophagy pathway (Hachez et al., 2014).

Recently, Yunfeng Shi et al. elucidated the mechanism of ABA accumulation in autophagy mutants under nitrogen starvation conditions from an epigenetic perspective. Under normal conditions, autophagy helps plants cope with nitrogen deficiency stress by maintaining genomic DNA methylation stability. When autophagy function is impaired, hypomethylation occurs in the *MYB101* gene promoter region under nitrogen-deficient conditions, leading to enhanced expression of this gene. *MYB101* promotes ABA synthesis, ultimately accelerating the plant senescence process (Shi et al., 2023). This discovery expands the new dimension of autophagy regulating ABA metabolism through epigenetic mechanisms.

In summary, autophagy participates in the regulation of plant ABA through diverse molecular mechanisms, exhibiting a pronounced context-dependent nature. A thorough elucidation of their interaction will provide crucial theoretical foundations for studying plant stress adaptation mechanisms and developing stress-resistant breeding strategies.

Overall, current studies indicate that autophagy acts as a complex and context-dependent regulator of ABA signaling, homeostasis, and downstream responses. By modulating ABA biosynthesis, receptor stability, signal transduction components, organelle quality control, and even epigenetic states, autophagy integrates hormonal cues with developmental programs and stress adaptation (**Figure 3**).

## 6 Salicylic acid

### 6.1 Regulation of autophagy by SA

SA and its related compounds play a crucial role in regulating plant autophagy, and the mechanisms are modulated by environmental conditions and key factors within signaling pathways.

Multiple studies have confirmed that SA or its analogues can directly or indirectly induce autophagy in plants. Treatment with BTH (benzo (1,2,3) thiadiazole-7-carbothioic acid), a synthetic SA analog, can induce autophagy (Yoshimoto et al., 2009). Moreover, SA or its methylated derivative MeSA also induces autophagy in *Arabidopsis* (Lijuan and Wenli, 2010). This induction effect was validated at the molecular level: SA treatment activated autophagy in *Arabidopsis*, leading to a significant increase in the levels of the autophagy marker ATG8-PE protein (Jeon et al., 2023).

The regulatory effects of SA on autophagy are conserved across different plant species and are influenced by nutritional conditions. In *Solanum tuberosum*, SA treatment significantly upregulates the expression of *StATG3*, *StATG9*, *StATG11*, *StATG13a*, and *StATG8-2.1* (Song et al., 2022). Promoter regions of *ATGs* in wheat and rice contain SA-related *cis*-acting elements (two in wheat and thirteen in rice), and five *ATGs* in rice show significant changes in expression levels following SA treatment (Yue et al., 2018; Xie et al., 2024). This suggests that the regulation of autophagy by the SA signaling pathway may be a conserved mechanism in plants. Notably, the outcome of SA-mediated autophagy regulation may be opposite depending on nutrient conditions. Under nutrient-rich conditions, SA, MeSA or BTH can induce autophagosome production in *Arabidopsis* seedlings (Yoshimoto et al., 2009; Lijuan and Wenli, 2010). However, under carbon starvation conditions, SA inhibits autophagy via NPR1 (Zhang et al., 2023a). This differential regulation indicates that the SA signaling pathway dynamically modulates autophagy activity in response to environmental conditions, thereby ensuring normal plant growth and stress adaptation across diverse physiological states.

At the physiological level, SA-induced autophagy is closely associated with plant stress resistance and senescence regulation. For example, in 4-week-old detached *Arabidopsis* leaves, low concentrations of exogenous SA (10 μM) can delay leaf senescence induced by exogenous methyl jasmonate (MeJA) (50 μM) by upregulating autophagy (Yin et al., 2020). Furthermore, SA-mediated autophagy plays a crucial role in plant disease resistance responses: SA-dependent autophagy occurs during pathogen infection in *Arabidopsis* (Zvereva et al., 2016; Shukla et al., 2022). Following CMV (cucumber mosaic virus) infection in *Arabidopsis*, the number of GFP-ATG8a tagged autophagosomes increased, and ATGs (such as ATG8a and NBR1) showed upregulation, indicating that autophagy was activated. However, in the background of the SA degradation gene NahG, CMV-induced autophagy was significantly attenuated, confirming that autophagy activation depends on SA signaling (Shukla et al., 2022). In wild-type *Arabidopsis*, submergence-induced autophagosome formation is blocked in *sid2* and *npr1–5* mutants, indicating that autophagy induction under submergence conditions depends on SA (Chen et al., 2015).

Members (NPR1, NPR3, NPR4) of the NPR family, core regulators of the SA signaling pathway, exhibit functional differences in autophagy regulation. Studies have shown that compared with Col-0, the *npr3 npr4* double mutant exhibits a more severe premature senescence phenotype and reduced autophagosome production (Wang et al., 2016), indicating that NPR3/4 positively regulate autophagy. In this study, researchers also found that NPR3 and NPR4 negatively regulate plant defense and senescence, whereas NPR1 exhibits opposite functions. NPR3 and NPR4 positively regulate programmed cell death (PCD), while NPR1 exerts an opposing effect (Wang et al., 2016). In addition, NPR3/4 and NPR1 also exert opposing effects in various other physiological processes (Zhang et al., 2006; Fu et al., 2012; Ding et al., 2018), Based on this, we can reasonably hypothesize: does NPR1 exert an opposite function to NPR3/NPR4 in autophagy regulation? In other words, could NPR1 act as a negative regulator of autophagy? Years later, another research result from the authors’ affiliated laboratory corroborated this hypothesis. Research findings by Baihong Zhang et al. indicate that endogenous or exogenous SA inhibits autophagy via NPR1 under carbon starvation stress, thereby accelerating leaf senescence in *Arabidopsis* (Zhang et al., 2023a). Accordingly, NPR1 inhibits autophagosome formation, whereas NPR3/4 appear to promote it, suggesting their opposing roles in autophagy regulation. Zheng Qing Fu et al. discovered that NPR3 and NPR4, as CUL3 adaptor proteins, promote NPR1 degradation. Consequently, *npr3*, *npr4*, and *npr3 npr4* plants exhibit elevated endogenous NPR1 levels (Fu et al., 2012). Consistent with the aforementioned findings, under dark treatment, *npr3 npr4* plants also exhibit a premature senescence phenotype and suppressed autophagy similar to those of NPR1-GFP (NPR1-overexpressing plants) (Wang et al., 2016; Zhang et al., 2023a). This implies that NPR3 and NPR4 may also induce autophagy by promoting NPR1 degradation, thereby delaying leaf senescence.

Overall, SA predominantly acts as an inducer of autophagy under many physiological and defense-related conditions, although this effect can be reversed under specific nutrient stresses. Mechanistic studies of this signaling pathway further suggest that members of the core SA signaling regulator NPR family play distinct roles in this process: NPR1 functions as a negative regulator of autophagy, whereas NPR3 and NPR4 promote autophagy. Together, these findings highlight the complexity of SA-mediated autophagy regulation and underscore the importance of functional divergence within the NPR family in fine-tuning autophagic activity (**Figure 4**).

### 6.2 Regulation of SA by autophagy

Regulation of autophagy on the SA pathway plays a crucial role in plant growth, immune responses, and stress adaptation. A common feature observed across multiple biological contexts is that autophagy deficiency frequently leads to elevated endogenous SA levels. This SA overaccumulation subsequently enhances SA-dependent physiological outputs, including premature senescence, immune activation and hypersensitivity under certain abiotic stresses.

#### 6.2.1 Regulation of SA and senescence by autophagy under normal growth conditions

Under normal growth conditions, autophagy maintains plant growth by suppressing the SA pathway, thereby delaying the process of premature senescence. Four-week-old *Arabidopsis* autophagy mutants begin exhibiting premature senescence phenotypes, with significantly higher expression levels of the senescence marker gene *SAG12* compared to wild-type plants. Analysis of phytohormone content revealed that endogenous SA levels in *atg5–1* were threefold higher than in wild-type *Arabidopsis*, with transcription levels of SA-responsive genes *PR1* and *PR2* also significantly upregulated. Genetic experiments confirmed that autophagy inhibits plant senescence by suppressing the SA pathway (Yoshimoto et al., 2009), establishing a core model in which autophagy delays senescence by limiting SA signaling.

This regulatory effect is not restricted to age-dependent senescence. ETH-induced senescence in the *atg2–2* mutant is also SA-dependent (Wang et al., 2011), suggesting that autophagy-mediated control of SA extends to phytohormone-triggered senescence programs. Notably, this regulatory model may exhibit tissue- or species-specificity. Unlike the case in *Arabidopsis* leaves, the SA content in rice anthers is not affected by autophagy deficiency (Kurusu et al., 2017).

#### 6.2.2 Regulation of the SA pathway by autophagy under pathogen infection conditions

Consistent with the general tendency toward SA overaccumulation in autophagy-defective plants, altered SA signaling is also central to immune responses during pathogen infection. However, in this context, the biological consequences of SA elevation depend strongly on the lifestyle of the invading pathogen. In plants, autophagy negatively regulates SA-dependent defense responses against biotrophic pathogens but positively regulates defense responses against necrotrophic pathogens.

Biotrophic pathogens obtain nutrients from living plant cells. They do not kill the host cells, which maintain a certain level of vitality throughout the infection. Effective plant defense against biotrophic pathogens is primarily mediated by the activation of defense-related responses regulated by PCD (programmed cell death) and SA-dependent pathways within the host (Glazebrook, 2005). Increasing evidence indicates that plants with autophagy defects exhibit significantly enhanced or even excessive immunity against biotrophic pathogen infections due to SA hyperactivation, suggesting autophagy negatively regulates plant immunity against biotrophic pathogens. For example, during infection by the half biotrophic pathogens *Pseudomonas syringae* pv tomato bacteria DC3000, excessive PCD associated with the excessive immunity of autophagy mutants induced by infection depends on SA signal transduction. In other words, autophagy inhibits excessive immunity-related PCD by suppressing signal transduction in the SA pathway, thereby confining PCD to the infection site (Yoshimoto et al., 2009; Lenz et al., 2011). Similar SA-dependent PCD have been observed in interactions with the biotrophic fungus *Golovinomyces cichoracearum* (Wang et al., 2011).

Viruses, which are also biotrophic pathogens, follow a comparable pattern. In *Arabidopsis*, autophagy alleviates symptoms during CMV infection by suppressing excessive SA activation, thereby maintaining post-infection plant fitness (Shukla et al., 2022). In soybean, plants with silenced *GmATG2*, *GmATG7*, or *GmATG5* exhibited significantly elevated SA content and more active expression of disease resistance-related genes (such as PR1), resulting in markedly enhanced resistance against biotrophic pathogens *Pseudomonas syringae* and *Soybean mosaic virus* (Hashimi et al., 2021, Hashimi et al., 2023, Hashimi et al., 2024).

Interestingly, this regulatory mode is reversed in some Rosaceae species, where autophagy enhances resistance to biotrophic pathogens by promoting SA accumulation (Sun et al., 2018, Sun et al., 2020), highlighting evolutionary divergence in autophagy–SA crosstalk.

Autophagy also promotes jasmonic acid (JA)-mediated plant defense against necrotrophic bacteria by inhibiting SA. Martine Rigault et al.’s study demonstrated that the *Arabidopsis atg2* mutant shows increased susceptibility to the necrotrophic bacterium *Dickeya dadantii*, and the underlying mechanism is that excessive SA signaling antagonizes the defensive effects of JA within the plant (Rigault et al., 2021). Similarly, in tomatoes, SlATG5 enhances fruit resistance to the necrotrophic fungus *Botrytis cinerea* by promoting autophagy, which suppresses SA signaling and allows activation of JA-mediated defense (Li et al., 2023).

Thus, during pathogen infection, autophagy does not simply suppress immunity; rather, it balances SA and JA signaling to prevent either insufficient or excessive defense, depending on pathogen lifestyle.

#### 6.2.3 Regulation of SA by autophagy under nutrient stress conditions

Under nutrient stress, autophagy performs both SA-dependent and SA-independent functions, indicating that SA overaccumulation is not the sole driver of all autophagy mutant phenotypes.

Several developmental defects in autophagy mutants occur independently of SA. For instance, research by Anne Guiboileau et al. indicates that *Arabidopsis atg5–1 sid2* and *atg5–2 NahG* exhibit nitrogen remobilization defects as severe as those in *atg5* single mutants, yet display significantly reduced early yellowing phenotypes. This suggests that the nitrogen remobilization defect caused by autophagy deficiency operates independently of SA levels, whereas the premature senescence phenotype depends on SA (Guiboileau et al., 2012) Likewise, the inhibition of primary root growth in *Arabidopsis* autophagy mutants induced by nitrogen starvation (Yoshimoto et al., 2009), dark-induced senescence (Yoshimoto et al., 2009) and carbon starvation-induced hypocotyl growth suppression (Avin-Wittenberg et al., 2015), all occur independently of SA, suggesting that autophagy supports nutrient recycling and growth through additional pathways.

Nevertheless, autophagy also influences SA metabolism under certain nutrient limitation. Metabolomics data indicate that under low nitrate conditions, the common precursors of SA and flavonoid compounds (shikimate and phenylalanine) in *Arabidopsis* autophagy mutants are preferentially allocated to the SA synthesis pathway, thereby depleting the biosynthesis of flavonoid compounds (Masclaux-Daubresse et al., 2014). Consistent with that, transcriptomic profiling revealed that SA biosynthesis genes (*ICS1* and *PBS3*), SA signaling pathway genes (*ENHANCED DISEASE SUSCEPTIBILITY 1* (*EDS1*), *PAD4*, *SALICYLIC ACID INDUCTION DEFICIENT 2* (*SID2*), and *NPR1-4*), and SA-responsive genes (PR1 and PR2) are upregulated in autophagy-deficient mutants (Masclaux-Daubresse et al., 2014). Regarding the specific node where autophagy regulates the SA pathway, transcriptomic data reveal that the *ANAC055* gene, described as a major negative regulator of SA biosynthesis, is significantly upregulated in autophagy mutants. This suggests that the defect in SA pathway regulation caused by autophagy deficiency may occur downstream or independently of the *ANAC055* regulatory node (Masclaux-Daubresse et al., 2014).

Recently, a study by Yunfeng Shi et al. revealed the mechanism underlying SA accumulation in autophagy mutants under nitrogen starvation from an epigenetic perspective. Under normal conditions, autophagy maintains the stability of DNA methylation to help plants cope with nitrogen deficiency. In the same nitrogen-deficient environment, when autophagy is defective, hypomethylation of the *PBS3* gene promoter enhances its expression. As a key enzyme in SA synthesis, this gene ultimately accelerates plant senescence (Shi et al., 2023).

#### 6.2.4 Regulation of the SA pathway by autophagy under abiotic stress

Similar to its role in development and immunity, SA also contributes to stress hypersensitivity in autophagy mutants under certain abiotic stresses. Autophagy counteracts ROS stress by suppressing SA. Hydrogen peroxide level measurements in autophagy mutants, SA-related mutants, and their double mutant combinations indicate that a portion of ROS accumulates in autophagy mutants in an SA-dependent manner (Yoshimoto et al., 2009). Experiments by Liang Chen et al. validated the aforementioned findings and perspectives: elevated ROS levels in *atg5–1* leaves were reduced in double mutants harboring *sid2* or *npr1-5* (Chen et al., 2015).

The sensitivity of *atg* mutants to submergence requires SA signal transduction. Under submergence stress, higher levels of SA accumulate in *atg* mutants, and the mRNA abundance of SA biosynthetic genes is also significantly higher than that in wild-type plants. The hypersensitive phenotype of *atg5–1* to submergence treatment is partially suppressed in the *atg5 sid2* double mutant, but fully suppressed in the *atg5 npr1* double mutant (Chen et al., 2015), demonstrating that autophagy regulates plant responses to waterlogging stress through SA signaling transduction.

#### 6.2.5 SAG may be cargo delivered from autophagy-associated ER to vacuoles

In addition to regulating SA biosynthesis and signaling, autophagy may also influence SA homeostasis at the level of intracellular transport. It is well known that SA is primarily modified by SA glucosyltransferase in the cytoplasm into SAG (SA O-β-glucoside), and accumulated in the vacuole (Vlot et al., 2009). Ivan Kulich et al. speculate that plants may utilize ER-derived autophagosome-like vesicles to transport SAG to vacuoles for storage or degradation (Kulich and Žárský, 2014).

Collectively, current evidence supports a unifying model in which autophagy functions as a buffering system that prevents excessive SA accumulation. When this buffering capacity is lost, SA levels rise across multiple contexts, but the physiological consequences diverge: promoting senescence during development, intensifying defense against biotrophic pathogens, weakening resistance to necrotrophs through JA antagonism, and increasing sensitivity to certain abiotic stresses. At the same time, species-specific rewiring and SA-independent functions of autophagy add further complexity to this regulatory network. Elucidating how autophagy modulates SA homeostasis under different biological scenarios will provide a theoretical basis for improving plant growth and disease resistance (**Figure 4**).

## 7 JA

### 7.1 Regulation of autophagy by JA

The JA signaling pathway plays a crucial role in the regulatory network of plant autophagy, with its regulatory function supported by experimental evidence across multiple plant species. In *Arabidopsis*, JA promotes petal abscission by inducing autophagy. Research by Yuki Furuta et al. has revealed the molecular mechanism of this process: JA is synthesized in stamens and transported to the base of petals, where it promotes degradation of JAZ proteins, inhibitors of JA signaling, thereby releasing MYC transcription factors. Liberated MYC binds to the promoter of target gene *ANAC102*, enhancing its expression. ANAC102 activates *ATGs* to promote autophagosome formation and degradation of cellular components, ultimately driving petal abscission (Furuta et al., 2024). In tomatoes, autophagy is suppressed in the JA receptor mutant tomato *jai1*, suggesting that autophagy may be promoted by JA signaling (Zhang et al., 2016a). Furthermore, analysis of gene promoters revealed the presence of two MeJA-related elements in the promoters of wheat ATGs (Yue et al., 2018). Thirty JA-responsive *cis*-acting elements are present in the promoters of rice *ATGs*, and five *ATGs* show significant changes in expression levels following JA treatment (Xie et al., 2024), indicating that the JA signaling pathway dynamically regulates autophagy processes by modulating the transcriptional activity of *ATGs* in response to environmental conditions, thereby ensuring normal plant growth and development.

These studies collectively support a model in which JA generally promotes autophagy. JA exerts this effect by relieving JAZ-mediated repression, thereby activating downstream transcription factors that regulate ATG expression, and possibly also through direct transcriptional activation of ATG genes. Through these mechanisms, JA signaling effectively integrates developmental cues and environmental signals with the autophagic machinery.

### 7.2 Regulation of JA by autophagy

Under normal growth conditions, the endogenous levels of JA and JA-Ile in 4-week-old *atg5–1* mutant *Arabidopsis* are approximately twice those of the wild type (Yoshimoto et al., 2009). However, observations of the premature senescence in autophagy mutants, JA-related mutants, and their double mutant combinations reveal that the premature senescence in autophagy mutants does not require an intact JA signaling pathway (Yoshimoto et al., 2009). Consistent with this, studies using *atg2–2* as experimental material reached the same conclusion (Wang et al., 2011). Meanwhile, transcriptomic data also revealed that genes involved in or responsive to the JA signaling pathway showed minimal differential expression in autophagy mutants (Masclaux-Daubresse et al., 2014).

In the condition of pathogen infection, autophagy plays a crucial role in the defense response regulated by JA. Necrotrophic pathogens kill host cells and obtain nutrients from necrotic tissues. They benefit from host cell death, thus evading restrictions imposed by cell death and SA-dependent defenses. JA-activated defense responses represent one of the key strategies plants employ to limit infection by necrotrophic pathogens (Glazebrook, 2005). Autophagy plays a key role in this defense strategy. Research findings by Zhibing Lai et al. indicate that autophagy suppresses the basal expression of the JA-regulated defense gene *PDF1.2* in healthy plants. However, upon infection by the necrotrophic fungal pathogen *Botrytis cinerea*, autophagy positively regulates the expression of *PDF1.2* in plants, and hypersensitivity of autophagy mutants to necrotrophic fungal pathogens is associated with reduced expression of the JA-regulated *PDF1.2* gene (Lai et al., 2011). Studies in certain species indicate that the susceptibility of autophagy mutants to necrotrophic pathogens is not directly caused by autophagy deficiency, but rather arises from the accumulation of SA in autophagy mutants antagonizing the defensive effects of JA (Rigault et al., 2021; Li et al., 2023).

In plant-pathogen interactions, autophagy exhibits diverse regulatory mechanisms for JA. In tomatoes, autophagy enhances defense against nematodes by promoting the degradation of JAM1/2/3, negative regulators of JA signaling (Zou et al., 2023). in *Eureka lemon*, the *ClBeclin1* and *ClAPX1* interact, with *ClBeclin1* specifically degrading *ClAPX1* (a JA inhibitor)via the autophagy pathway (Wang et al., 2023). Upon degradation of ClAPX1, its inhibitory effect on JA synthesis is lost, leading to JA accumulation within citrus plants. This accumulation subsequently activates the plant’s defense response against CYVCV (citrus yellow vein clearing virus) (Wang et al., 2024a).

Some viruses hijack autophagy to disrupt the plant’s JA defense system, thereby facilitating their own replication. P10, a rice black-streaked dwarf virus capsid protein, directly interacts with OsFAD7 (a key enzyme in JA precursor synthesis) and promotes its autophagic degradation via the AIM (ATG8 interaction motif), leading to reduced JA content and impaired rice defense functions. The study also found that SP10, a capsid protein from another virus in the same genus, southern rice black-streaked dwarf virus, degrades OsFAD7 using a similar mechanism, suggesting this strategy may be common among related viruses (Liu et al., 2025). Furthermore, Turnip mosaic virus (TuMV) has evolved a strategy to counteract host immune responses by promoting the degradation of a positive regulator of JA biosynthesis. The TuMV P1 protein interacts with the JA biosynthesis positive regulators cpSRP54 and mediates its degradation through both the proteasome and autophagy pathways. As a result, cpSRP54 levels are reduced in TuMV-infected *Nicotiana benthamiana*, leading to suppression of JA biosynthesis and enhanced viral infection (Ji et al., 2021). Consistent with this, TuMV infection has been shown to reprogram autophagy-related genes such as *NBR1*, thereby stabilizing viral proteins and enhancing systemic accumulation. In addition, TuMV suppresses the expression of *LOX1* and *LOX2*, two enzymes involved in JA biosynthesis, further weakening host defense responses (Bera et al., 2022). Together, these findings underscore that TuMV exploits autophagy alongside the direct suppression of JA synthesis.

**Figure 5:**
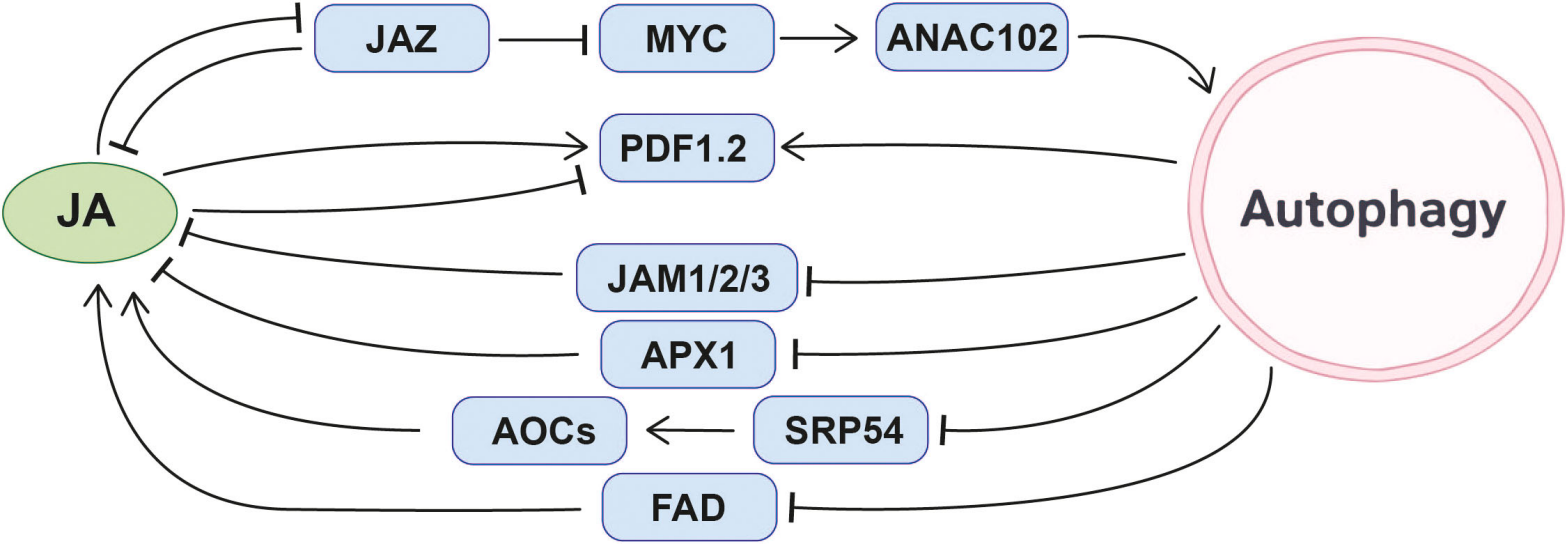
Molecular interplay mechanisms between jasmonic acid (JA) and autophagy.

In summary, autophagy modulates JA levels and signaling outputs in a highly context-dependent manner, contributing to defense against necrotrophic pathogens while also being targeted by viruses to suppress JA-mediated immunity. This highlights autophagy as both a regulator and a target within JA-associated defense networks (**Figure 5**).

## 8 ETH

### 8.1 Regulation of autophagy by ETH

Numerous experimental results have shown that exogenous application of ETH can directly activate autophagy. Following treatment of 4-day-old *Arabidopsis* with the ETH precursor ACC(1-aminocyclopropane-1-carboxylic acid), autophagy activity was significantly enhanced and autophagosome numbers increased, indicating that ETH itself induces autophagy (Kumaran et al., 2025). In *Ipomoea nil* petals, ETH treatment upregulates *InATG4b* expression and accelerates floral senescence (Yamada et al., 2009). In petunia corollas, transcript abundance of multiple *ATGs* increased after 4 hours of ETH treatment (Quijia Pillajo et al., 2018).

Endogenous ETH produced by different plant species under various physiological conditions responds to external environmental changes by inducing autophagy. In soybean, starvation stress may regulate the expression of *ATGs* (such as *GmATG8i* and *GmATG4*) through the ETH signaling pathway (including *GmACCS*, *GmERF*, *Ein3*, etc.), thereby promoting autophagy. This process helps soybeans degrade and recycle intracellular components during starvation to cope with nutrient-deficient environments (Okuda et al., 2011). In petunia petals, pollination leads to increased endogenous ETH levels, and this elevated ETH induces the expression of the *PhATG8* homolog. Similarly, exogenous ETH treatment of unpollinated petunias also induces the expression of the *PhATG8* homolog (Shibuya et al., 2013). In *Solanum lycopersicum*, under drought stress, tomatoes produce ETH, which induces ERF5 (Ethylene response factor 5) to bind to the *ATG8d/ATG18h* promoter. This promotes autophagy to recycle nutrients and maintain cellular homeostasis, thereby enhancing tomato drought tolerance (Zhu et al., 2018). Some experts speculate that ETH plays a critical role in inducing autophagy and promoting ROS amelioration, thereby contributing to enhanced survival rates during flooding, hypoxia, and reoxygenation stress (Hartman et al., 2021). Research by Zheng Qiwei et al. confirmed this hypothesis: during the early stages of submergence stress, ETH levels significantly increased and induced autophagy activity. Autophagy primarily eliminated damaged mitochondria, the main source of ROS, helping *Arabidopsis* root cells reduce ROS damage and improve survival rates (Zheng et al., 2023). Additionally, ETH is a phytohormone crucial for citrus fruit coloration, promoting the expression of the ETH response factor CsERF061. CsERF061 directly binds to the promoter of *CsATG8h* and activates its expression, thereby promoting autophagy and fruit coloration. This finding indicates that ETH and autophagy work together to promote citrus coloration (Guo et al., 2025b).

ETH-mediated autophagy regulation can also be achieved through transcriptional regulation. Y1H (Yeast 1-hybrid) screening results revealed that TFs (transcription factors) from the AP2-EREBP (APETALA2/ethylene-responsive element binding proteins) family were enriched in both *ATG8a* and *ATG8h* screens (Wang et al., 2020), indicating that ETH signaling can regulate the expression of *Arabidopsis ATGs*. Promoter analysis revealed several ETH-responsive TFs binding elements within the *ATG* promoters of wheat and *Arabidopsis* (Yue et al., 2018; Wang et al., 2020). Sixteen ETH-responsive *cis*-acting elements are present in the promoters of rice *ATGs* (Xie et al., 2024), further suggesting that ETH signaling may dynamically regulate autophagy activity through the binding of transcription factors to *ATG* promoters in response to environmental conditions, thereby influencing plant survival under stress.

Overall, ETH acts as a positive regulator of autophagy across multiple species and stress conditions, largely through transcriptional activation of ATGs and ROS-related signaling. ETH-induced autophagy plays key roles in senescence, hypoxia tolerance, stress acclimation, and developmental transitions.

### 8.2 Regulation of ETH by autophagy

Research indicates that autophagy suppresses exogenous ETH-induced premature senescence in *Arabidopsis*, as evidenced by *atg2–2* plants exposed to ETH exhibiting accelerated senescence compared to wild-type plants (Wang et al., 2011). However, ETH-related pathways do not participate in the premature senescence of autophagy mutants. Observation of the senescence phenotypes of the autophagy mutant *atg5-1*, ETH-related mutants, and their double mutant combinations confirmed that the premature senescence of autophagy mutants does not require an intact ETH signaling pathway (Yoshimoto et al., 2009). Consistent with this, studies using *atg2–2* as experimental material have further validated this conclusion (Wang et al., 2011).

ETH-related pathways may participate in other physiological processes besides senescence regulation. A study revealed that methionine levels are significantly elevated in autophagy mutants, and the expression of numerous genes involved in methionine synthesis and salvage pathways is markedly increased. Since methionine serves as a precursor for ETH synthesis in *Arabidopsis*, it is hypothesized that autophagy mutants may exhibit heightened ETH synthesis activity (Masclaux-Daubresse et al., 2014). According to a study on autophagy and the hypoxic response in *Arabidopsis*, several genes associated with ETH biosynthesis and signal transduction exhibited lower relative expression levels in autophagy mutants following submersion treatment compared to wild-type plants, indicating that autophagy deficiency impairs ETH-related pathways (Chen et al., 2015). Studies using petunia as experimental material demonstrate that silencing *ATGs* suppresses the expression of *PhACS*, which is a ETH biosynthesis genes (Lin and Jones, 2021). The following year, another related academic report published by the same research group demonstrated that *PhACS* and *PhACO1* expression was upregulated in *PhATG6*-knockout (KO) lines, suggesting that *PhATG6* may delay petal senescence by inhibiting ETH synthesis (Lin and Jones, 2022). The absence of *ATGs* in these two reports had opposite effects on *PhACS* expression. This discrepancy may stem from the use of different petunia cultivars in the two experiments, or it could be due to the failure to detect another ACS member in petunia. The relationship between ETH signal transduction and autophagy remains shrouded in numerous unresolved mechanisms, requiring further investigation to elucidate them.

Recent research has further expanded our understanding of the relationship between autophagy and ETH. Research by Anna Coll et al. has revealed a phenomenon where a pathogen manipulates autophagy for its own benefit. By inhibiting autophagy, the pathogen impedes the degradation of the ETH response factor StPti5, thereby promoting infection (Coll et al., 2024). Another study indicates that autophagy acts as a “braking mechanism” in tomato fruit ripening: it suppresses the production and action of ETH, thereby delaying fruit maturation. When autophagy function weakens, ETH is no longer effectively suppressed, leading to accelerated fruit ripening (Kumaran et al., 2025).

Current evidence indicates that autophagy can influence ETH biosynthesis and signaling in a species- and tissue-dependent manner, particularly during senescence and stress responses. However, the underlying regulatory mechanisms remain unclear, highlighting the need for further mechanistic investigation.

## 9 Strigolactone

SL is a recently discovered class of terpenoid phytohormone that plays a crucial role in plant development, particularly in inhibiting shoot branching. However, current research on the association between SL and plant autophagy remains limited, and the underlying mechanisms require further investigation.

Recent studies in tomatoes have provided important clues for revealing new functions of SL in autophagy. After exogenous application of a synthetic SL analog, autophagic activity in tomato is significantly enhanced, and its cold tolerance is remarkably improved (Chi et al., 2025). Further studies have shown that under cold stress, the endogenous SL synthesized by tomato increases. SL induces the accumulation of the transcription factor HY5, which directly binds to the promoter of the autophagy-related gene *ATG18a*, activates its expression, thereby promoting autophagosome formation, enhancing autophagic function, facilitating the clearance of cold-induced damaged proteins, and ultimately improving tomato’s cold tolerance (Chi et al., 2025).Although research remains limited, emerging evidence suggests that SL positively regulates autophagy under stress conditions, particularly during cold stress in tomato. This points to a previously underappreciated role of SL in linking environmental adaptation with cellular autophagy.

## 10 Brassinosteroid

### 10.1 Regulation of autophagy by BR

In plant physiological processes, the regulation of autophagy by the BR signaling pathway exhibits complex mechanisms, and its effects vary depending on plant species and stress type. The core mechanism of BR signal transduction involves key receptors and regulatory factors. BAK1, as a BR co-receptor for BRI1, has been demonstrated to regulate BR signaling (Li et al., 2002; Nam and Li, 2002). A recent study indicates that BAK1 influences autophagy activity by regulating the phosphorylation status of ATG18a: BAK1-mediated phosphorylation of ATG18a suppresses autophagy and reduces disease resistance, whereas dephosphorylation of ATG18a activates autophagy, aiding plants in resisting necrotrophic pathogens (Zhang et al., 2021). Ching-Yi Liao et al. found that blocking BR biosynthesis or signaling leads to sustained upregulation of autophagy, while enhancing BR pathway activity downregulates autophagy. Furthermore, their research revealed an additional molecular mechanism by which BR regulates autophagy. BR inhibits GSK3-like kinase BIN2, which acts upstream of the TOR complex and suppresses its activity, thereby promoting autophagy. Their research further revealed that BIN2 influences downstream signaling pathways controlling plant nutrient cycling by phosphorylating the substrate-recruitment subunit within the TOR complex (Liao et al., 2023).

The regulatory effect of BR on autophagy varies among plant species. Consistent with Arabidopsis, EBR (24-epibrassinolide), a highly active synthetic analog of the BR, treatment in peach trees alleviated drought stress damage to peach leaves, reduced *PpATGs* expression levels, and decreased the number of autophagosomes (Wang et al., 2019a). However, in tomatoes and grapevines, BR positively regulates autophagy. In *Solanum lycopersicum*, BR signaling primarily exerts an upregulating effect on autophagy. BZR1 acts as a transcription factor for the *SlATG2* and *SlATG6*, regulating their transcription. BZR1-dependent BR signaling can upregulate *SlATG*s expression and autophagosome formation, enabling autophagy to selectively degrade ubiquitinated proteins under nitrogen starvation conditions and enhancing tomato tolerance to nutrient deficiency (Wang et al., 2019b). Additionally, BZR1 also acts as a transcription factor for *SlNBR1* and several *SlATGs* genes, activating their transcription and thereby promoting autophagy to enhance tomato cold tolerance (Chi et al., 2020). In grapevines, BR mitigates the detrimental effects of drought stress by promoting autophagy activity, thereby facilitating the degradation of damaged chloroplasts (Zeng et al., 2022). Alternatively, by activating VvBZR1, a TF binds to the promoter of the *VvATG18a*, thereby initiating its expression and enhancing autophagy activity. This process assists grapevines in eliminating invading gray mold fungi and improving disease resistance (Zhou et al., 2025).

In summary, BR exerts species- and context-dependent effects on autophagy, functioning either as a positive or negative regulator. At the mechanistic level, BR signaling modulates autophagy through multiple molecular nodes, including phosphorylation of autophagy-related proteins as well as transcriptional activation of NBR1 and various ATG genes. Together, these regulatory routes enable BR to fine-tune autophagic activity in coordination with developmental and environmental cues.

### 10.2 Regulation of BR by autophagy

BES1 (BRI1-EMS-SUPPRESSOR 1) and BZR1 (BRASSINAZOLE RESISTANT 1) are not only key TFs in the BR signaling pathway, but also serve as central hubs integrating multiple signals to regulate plant development and environmental adaptation (Wang et al., 2014). Several reports have revealed the mechanism that plants degrade BES1 and BZR1 through autophagy during sucrose starvation to reduce growth. BES1 and BZR1 are degradation targets of plant autophagy, which coordinates plant growth and sugar starvation stress by regulating the levels of BES1 and BZR1. Specifically, sugar signaling activates the TOR pathway to promote the accumulation of BES1 and BZR1, thereby enhancing plant growth. Conversely, sugar starvation-induced TOR inactivation promotes autophagy-mediated degradation of BES1 and BZR1, subsequently inhibiting plant growth. This mechanism balances the levels of available carbon and growth in *Arabidopsis* under varying sugar concentrations (Zhang et al., 2016b; Nolan et al., 2017; Wang et al., 2021; Zhang et al., 2023b). By the way, in addition to the autophagy pathway, BES1 and BZR1 are also degraded via the proteasome pathway (Wang et al., 2013b; Kim et al., 2014; Yang et al., 2017; Park et al., 2022). Additionally, multi-omics data reveals that BR and TOR synergistically regulate growth and stress responses through shared downstream molecules: TOR may promote growth by stabilizing key TFs in the BR pathway, such as BES1, while BIN2 may influence nutrient sensing by inhibiting TOR-associated kinases. This cross-talk enables plants to flexibly adjust growth strategies based on phytohormone and nutritional conditions: under nutrient-rich conditions, TOR activation enhances the BR pathway to promote growth; under stress, both BR and TOR signaling diminish, activating autophagy to recycle resources (Montes et al., 2022).

The regulation of BR by autophagy is also reflected in its impact on BR synthesis. Research has found that genes associated with BR synthesis are generally downregulated in *atg5* (Song et al., 2024), suggesting that autophagy may participate in regulating BR synthesis through certain pathways.

Together, these studies demonstrate that autophagy directly modulates BR signaling output by degrading key transcription factors such as BES1 and BZR1, thereby coordinating plant growth with carbon and energy availability. This highlights autophagy as an important feedback regulator within the BR pathway.

## 11 Melatonin

Melatonin is an indole molecule known for inducing tolerance in plants against various stresses. Reports on multiple plant species indicate that melatonin can drive plant autophagy under stress conditions. In *Arabidopsis*, melatonin-treated plants accumulated more autophagosomes and exhibited higher expression of *AtATG8* subtypes under methyl viologen-induced oxidative stress, thereby reducing root damage. This demonstrates melatonin’s role in alleviating oxidative stress (Wang et al., 2015). In tomatoes, melatonin enhances heat tolerance by activating autophagy to clear abnormal proteins, thereby reducing protein damage caused by high temperatures (Xu et al., 2016), and alleviating high temperature-Induced pollen abortion (Qi et al., 2018). After introducing *MsSNAT*, a gene involved in melatonin biosynthesis in alfalfa, into *Arabidopsis*, the melatonin production significantly increased, autophagic activity was enhanced, and the tolerance of *Arabidopsis* to salt stress was improved (Zhao et al., 2019). In pear trees, exogenous melatonin application enhances resistance to *Botryosphaeria dothidea* by increasing autophagy activity (Wang et al., 2022b). In drought-sensitive cotton (*Gossypium hirsutum* L.), drought treatment reduced the expression levels of *ATG8c/8f*. However, when melatonin treatment was applied concurrently with drought, *ATG8c/8f* expression significantly increased, ATG8-PE protein content rose, and plants exhibited enhanced drought tolerance (Supriya et al., 2022, Supriya et al., 2024; Dake et al., 2025). In cassava, there is a positive feedback regulation between melatonin and autophagy. Exogenous melatonin administration or overexpression of melatonin synthase induced the expression of multiple *ATGs* and enhanced autophagy activity, manifested as an increase in autophagosome numbers. Overexpression of *MeATG8s* increases melatonin synthase protein levels, thereby elevating melatonin content in cassava. Conversely, silencing these *ATGs* reduces both melatonin content and autophagy activity. In this study, the authors also discovered that three melatonin synthases (MeTDC2, MeASMT2, MeASMT3) interact with MeATG8b/8c/8e both *in vivo* and *in vitro*. This is the first study demonstrating a direct link between melatonin and autophagy in cassava (Wei et al., 2020).

Under certain conditions, melatonin also exerts an inhibitory effect on autophagy. In apple leaves, nearly all *ATGs* showed significantly increased expression during the senescence process, but melatonin treatment significantly reduced *ATGs* expression, indicating that melatonin suppresses autophagy. The authors propose that this inhibitory effect alleviates the degradation of proteins and organelles, thereby delaying leaf senescence (Wang et al., 2013a).

Taken together, current evidence suggests that melatonin and autophagy generally promote each other, although in certain developmental contexts, such as leaf senescence in apple, melatonin may suppress autophagy. These findings broaden our understanding of the coordination between plant autophagy signaling and melatonin biosynthesis, and may provide a theoretical basis for future strategies that exploit their synergistic effects to enhance plant stress resistance.

## 12 Discussions and future perspectives

Existing research has revealed extensive bidirectional interactions between autophagy and phytohormone pathways. Rather than functioning as isolated signaling events, these interactions position autophagy as a central regulatory module within the plant hormone network. A key concept is that autophagy not only responds to hormonal cues, but also shapes hormone homeostasis, modulates signaling amplitude, and integrates multiple hormonal inputs to balance plant growth, development, and stress adaptation.

A unifying concept emerging from current studies is that autophagy contributes to maintaining phytohormone homeostasis. By regulating the turnover of hormone-associated proteins, signaling components, and even organelles involved in phytohormone metabolism, autophagy influences phytohormone abundance, spatial distribution, and turnover dynamics. Through these processes, autophagy acts as a buffering system that prevents excessive accumulation or prolonged persistence of hormonal signals, thereby preserving developmental stability while allowing flexible responses to environmental change.

Beyond regulating hormone abundance, autophagy also controls the intensity and persistence of hormonal signaling. Many autophagy-deficient mutants display hypersensitive or prolonged hormone responses, indicating that autophagy functions as a negative feedback mechanism to dampen overactive signaling pathways. This phenomenon is particularly evident in stress-related hormones. Rather than acting as a simple inhibitor, autophagy appears to establish signaling thresholds that ensure hormonal outputs remain proportional to developmental cues and stress intensity. This modulation of signaling amplitude helps prevent maladaptive outcomes such as excessive defense activation, runaway cell death, or premature senescence.

Plant growth and stress adaptation rely on synergistical or antagonistical among multiple phytohormones rather than single phytohormone pathways. However, how these signals converge on autophagy is only beginning to be understood. Existing observations support a model in which autophagy acts as a decision-making node, integrating hormonal, metabolic, and stress-derived signals to allocate cellular resources between growth, defense, and survival. Understanding how multiple phytohormones collectively regulate autophagy, and how autophagy in turn reshapes phytohormone crosstalk networks, represents an important frontier for future research.

Expanding the physiological and environmental contexts of study will further clarify the breadth of autophagy–hormone interactions. Many developmental transitions and tissue-specific processes remain insufficiently explored, and phytohormone–autophagy relationships often display strong context dependency. Investigating these interactions under combined stresses and across diverse plant species will help distinguish conserved regulatory principles from lineage-specific adaptations.

From an applied perspective, the bidirectional regulatory relationship between autophagy and phytohormones offers promising opportunities for crop improvement. Genetic engineering and genome editing of key genes in the autophagy-phytohormone crosstalk network in plants may enhance crop resilience to drought, salinity, nutrient limitation and pathogen attack. Beyond genetic approaches, increasing attention is being directed toward other environmentally friendly strategies, such as the use of natural metabolites and biostimulants, which have demonstrated considerable potential in promoting sustainable agricultural practices and improving plant stress tolerance (Parizad and Bera, 2023). These natural or environmentally friendly interventions may influence endogenous regulatory networks, including phytohormone signaling and autophagy, which are key stress-responsive pathways. Integrating such eco-friendly strategies with molecular insights into autophagy regulation could provide innovative avenues for strengthening intrinsic plant defense systems during crop improvement. These perspectives are particularly relevant in the context of organic and sustainable agriculture, where enhancing crop resilience through internal regulatory networks mediated by autophagy and phytohormones rather than external chemical inputs is a key objective. Beyond crop improvement, the regulatory crosstalk between autophagy and phytohormones also holds great potential in medicinal plant research. Further studies may explore how the interaction between autophagy and phytohormones influences the biosynthesis of secondary metabolites. Manipulating these regulatory networks could offer new strategies to enhance the accumulation of bioactive compounds or pharmaceutical intermediates, thereby increasing the medicinal and economic value of such plants.
