# Supplementary material for: Autophagy-phytohormone crosstalk: a dual-regulation axis in plant development
Source: Front Plant Sci. 2026 Feb 26;17:1781813. doi: 10.3389/fpls.2026.1781813 (PMC12979181; doi:10.3389/fpls.2026.1781813)
Supplement: Supplementary file 1 [file Table1.docx]

Table S1: Regulation of phytohormones by Autophagy.

| Phytohormones | Category | Subfield | Species | Line | Function | Reference |
| --- | --- | --- | --- | --- | --- | --- |
| Auxin | Growth and development | Root | *Arabidopsis* | *atg5-1，atg7-3* | Autophagy plays a critical role in glucose-mediated regulation of root meristem activity by maintaining cellular homeostasis of auxin. | [31] |
| Auxin |  |  | *Arabidopsis* | *atg5-1, atg7-2* | Autophagy enhances root sensitivity to sucrose excess | [32] |
| Auxin |  |  | *Arabidopsis* | *atg2-1, atg5-1* | Autophagy regulates the periodic fluctuations and quantitative balance of the auxin response factor ARF7, thereby influencing the auxin signaling pathway's regulation of lateral root formation. | [33] |
| Auxin |  | Senescence | maize | Si-287，Si-144 | The dynamic balance of *ZmATG18b* and auxin synthesis-related gene *ZmGH3.8* plays a crucial role in leaf senescence. | [36] |
| Auxin | Stress | nutrient | *Arabidopsis* | *AtPUB9* （SALK_020751），*AtARK2* （SAIL_594_A06） | Under Pi starvation, autophagy promotes auxin accumulation in lateral root primordia by degrading auxin accumulation inhibitors, thereby inducing increased lateral root formation as a response to Pi starvation. | [34,35] |
| Auxin |  | heat | *Arabidopsis* | *atg7* | In heat-sensitive *fes1a*, autophagy indirectly suppresses auxin synthesis by degrading peroxidases, thereby weakening the plants' heat tolerance. | [37] |
| Auxin |  |  | *Arabidopsis* | GFP-ATG8a， mCherry-ATG8e， *atg5-1* | Activation of autophagy under heat stress enhances heat dissipation by promoting hypocotyl elongation through regulating auxin distribution. | [38] |
| CK | Growth and development | roots | *Arabidopsis* | GFP-AtAtg8f-HA | Autophagy influences CK-mediated regulation of root architecture | [40] |
| CK | Pathway | long-distance signal transduction | *Arabidopsis* | GFP-AtAtg8f-HA | Autophagy negatively regulates CK-mediated root–shoot communication. | [40] |
| CK |  | Signal pathway | *Arabidopsis* | *atg5* | *atg5* exhibits deficiencies in perception and response within CK. | [43] |
| CK |  |  | *Arabidopsis* | *atg5* | Autophagy relieves the inhibition of CK signaling by degrading type-A ARR proteins. | [41] |
| CK |  | Endogenous levels of metabolites | *Oryza sativa* L. | *Osatg7-1* | Autophagy influences CK metabolism and endogenous levels in anthers. | [42] |
| GAs | Growth and development | flower | *Oryza sativa* L. | *Osatg7-1* | Autophagy promotes pollen development by positively regulating GAs content in anthers. | [42] |
| ABA | Growth and development | senescence | *Arabidopsis* | *atg5-1* | MYB101 hypomethylation in autophagy mutants under nitrogen deficiency enhances ABA biosynthesis and accelerates senescence. | [69] |
| ABA |  | Seed germination, stomatal movement, lateral root development | *Arabidopsis* | NBR1-OX, nbr1-KO | The selective autophagy cargo receptor NBR1 can bind to the key ABA signaling transcription factors ABI3, ABI4, and ABI5. | [53] |
| ABA | Stress | drought | *Musa acuminata* | MaATG8f-OX | MaATG8f-OX *Arabidopsis* showed elevated leaf ABA levels and improved drought resistance. | [47] |
| ABA |  |  | *Malus domestica* | *MdATG10-*OE apple lines | *MdATG10*-OE apple lines exhibited reduced ABA pathway gene expression, sustained stomatal opening and improved drought tolerance. | [54] |
| ABA | Pathway | Endogenous levels of metabolites | *Solanum lycopersicum* L. | *SlATG7*-RNAi | Autophagy-deficient plants exhibit elevated ABA levels in tomato fruits during the late ripening stage. | [55] |
| ABA |  | Signal pathway | *Arabidopsis* | Col-0, Wassilewskija | Autophagy may degrade SDIRIP1 to regulate ABI5-mediated ABA signaling | [59] |
| ABA |  |  | *Arabidopsis* | *atg7-3* | FREE1 and HA-VPS23A stability increased in *atg7-3*, and ConA inhibited their degradation. | [58] |
| ABA |  |  | *Arabidopsis* | GFP-AtATG8e，*atg5* | Selective autophagy degrades ABA-induced protein At-TSPO. | [65,66] |
| ABA |  |  | *Arabidopsis* | *atg5* | Autophagy degrades the ABA-induced PIP2;7-TSPO complex. | [68] |
| ABA |  |  | *Arabidopsis* | *AtNBR1*-TAP | *ABA-responsive genes were upregulated in AtNBR1-TAP，which exhibited ABA-sensitive phenotypes* | [53] |
| ABA |  |  | *Arabidopsis* | *atg2-5* | Autophagy modulates ABA-mediated stomatal function via ROS. | [61] |
| SA | Growth and development | senescence | *Arabidopsis* | *atg5-1* | Autophagy relies on NPR1 to regulate SA homeostasis and suppress premature senescence. | [70] |
| SA |  |  | *Arabidopsis* | *atg2-2* | Autophagy suppresses premature senescence via the SA pathway. | [83] |
| SA |  |  | *Arabidopsis* | *atg5-1* | PBS3 hypomethylation in autophagy mutants under nitrogen deficiency enhances SA biosynthesis and accelerates senescence. | [69] |
| SA | Stress | submergence | *Arabidopsis* | *atg5-1* | Autophagy enhances submergence tolerance via SA reduction. | [78] |
| SA |  | pathogen | *Arabidopsis* | *atg5-1* | Autophagy relies on NPR1 signaling to inhibit excessive immunity-related PCD by reducing SA. | [70] |
| SA |  |  | *Arabidopsis* | *atg5* | Autophagy attenuates SA responses to alleviates post-infection symptoms and maintain plant fitness. | [76] |
| SA |  |  | *Arabidopsis* | *atg2-2* | Autophagy partially inhibits fungus-induced cell death by suppressing the SA pathway. | [83] |
| SA |  |  | *Glycine max* | *GmATG2-*silenced | Autophagy downregulates SA to impair plant resistance to *Pseudomonas syringae.* | [86] |
| SA |  |  | *Glycine max* | *GmATG7-*silenced | Autophagy downregulates SA to impair plant resistance to *Pseudomonas syringae* and soybean mosaic virus. | [87] |
| SA |  |  | *Glycine max* | *GmATG5-*silenced | Autophagy downregulates SA to impair plant resistance to *Pseudomonas syringae.* | [88] |
| SA |  |  | *Solanum lycopersicum* | *Slatg5* | Autophagy inhibits the SA signaling pathway to enhance plant resistance to *Botrytis cinerea*. | [92] |
| SA |  |  | *Malus × domestica* cv. Royal Gala | OE of *MdATG18a* | Autophagy upregulates SA to boost plant resistance to *Diplocarpon mali*. | [89,90] |
| SA |  | pathogen | *Arabidopsis* | *atg5, atg10* | Autophagy negatively regulates SA signaling to inhibit excessive immune responses. | [85] |
| SA | Pathway | Signal pathway | *Arabidopsis* | *atg5-1* | High ROS in autophagy mutants relies on SA pathway. | [70,78] |
| SA |  | Endogenous levels of metabolites | *Arabidopsis* | *atg5-1* | SA and its two precursors, shikimate and phenylalanine, are accumulated in autophagy mutants. | [43] |
| SA |  |  | *Arabidopsis* | *atg5, atg9* | Genes related to SA biosynthesis, signaling and response are upregulated in autophagy mutants. | [43] |
| JA | Stress | Pest infestation | Solanum lycopersicum | *atg4、atg6、atg7和atg10* | Root-knot nematode infection activates plant autophagy, which degrades JAM, relieves the inhibition of ERF1-MED25 complex, and thus activates insect-resistant genes; ERF1 can positively regulate autophagy, forming an insect-resistant defense cycle. | [99] |
| JA |  | pathogen | Nicotiana benthamiana | silencing *ATG5 or ATG7* | Turnip mosaic virus induces autophagic degradation of cpSRP54, which mediates allene oxide cyclases transport to the thylakoid membrane. | [103] |
| JA |  |  | rice cultivar ZH11 | *OsATG8b-GFP* | Rice black-streaked dwarf virus coat protein P10 mediate fatty acid desaturases autophagic degradation, reducing rice JA content and impairing defense. | [102] |
| JA |  |  | *Arabidopsis* | *atg5、atg7和atg18a* | Autophagy inhibits the basal expression of PDF1.2 in healthy plants, but positively regulates its induction in plants infected by necrotrophic pathogens. | [98] |
| JA |  |  | *Solanum lycopersicum* | *Slatg5* | The JA signaling pathway is downregulated in *Slatg5* mutants after *Botrytis cinerea* infection. | [92] |
| JA |  |  | Eureka lemon | OE-ClBeclin1 | Autophagy degrades ClAPX1, relieves JA inhibition, JA accumulation enhances antiviral defense | [101] |
| JA | Pathway | Endogenous levels of metabolites | *Arabidopsis* | *atg5-1* | JA and JA-Ile levels in autophagy mutants are double those of the wild type. | [70] |
| ETH | Growth and development | Fruit ripening | *Solanum lycopersicum* L. | *ATG4*-RNAi | Autophagy may delay fruit ripening by inhibiting ETH. | [104] |
| ETH |  | senescence | *Arabidopsis* | *atg2-2* | Autophagy can inhibit exogenous ETH-induced premature senescence. | [83] |
| ETH | Stress | pathogen | *Solanum tuberosum* L. |  | Pathogens inhibit autophagy to block the degradation of ETH-responsive factor StPti5, thereby promoting infection. | [115] |
| ETH | Pathway | Endogenous levels of metabolites | *Arabidopsis* | *atg5, atg9, ATG18a* RNA interference lines | Methionine, the precursor of ETH biosynthesis, accumulates significantly in autophagy-deficient mutants, with highly active metabolism and salvage pathways. | [43] |
| ETH |  | Gene expression regulation | *Arabidopsis* | *atg5-1，atg7-3* | After submergence treatment, the relative expression levels of genes related to ETH pathway in autophagy mutants are significantly lower than those in wild-type plants. | [78] |
| ETH |  |  | Petunia | *PhATG6*- and *PhPI3K*-silenced petunias | *ATG* silencing inhibits ETH gene expression. | [113] |
| ETH |  |  | Petunia | *PhATG6-*KO | *PhACS* and *PhACO1* are upregulated in *PhATG6*-KO. | [114] |
| BR | Stress | nutrient | *Arabidopsis* | *atg5-1, atg7-2* | Autophagy coordinates carbon availability and plant growth by regulating the degradation of two BR-related proteins. | [127-129] |
| BR | Pathway | Gene expression regulation | *Arabidopsis* | *atg5* | Genes related to BR biosynthesis are generally downregulated in *atg5*. | [136] |
| melatonin | Pathway | Endogenous levels of metabolites | cassava | 35S::GFP-*MeATG8s*, pTRV-*MeATG8s* | *MeATG8s* overexpression increases the protein level of melatonin synthase; *MeATG8s* silencing reduces that. | [145] |

Table S2: Regulation of Autophagy by exogenous phytohormones.

| Phytohormones | Species | Growth stage | Treatment | Effect | Reference |
| --- | --- | --- | --- | --- | --- |
| Auxin | *Arabidopsis* | 7day(d) | 20 nM NAA for 6-8 h | Auxin blocks autophagy activation by activating TOR. | [21] |
| Auxin | *Arabidopsis* | 7 d | 20 nM NAA for 6 h | The basal autophagy level of GFP-ATG8e seedlings is lower than that of the control group after NAA treatment. | [27] |
| CK | *Arabidopsis* | 5 d | 100 ng/mL Zeatin for 10 d | Zeatin treatment significantly reduces autophagosomes in the vacuoles of root epidermal cells. | [40] |
| CK | *Arabidopsis* | 5 d | 100 ng/mL Zeatin for 10 d | Zeatin treatment induces the formation of GFP-AtATG8f-containing fluorescent structures near the deep vascular system of roots. | [40] |
| CK | *Brassica rapa* subsp. *chinensis* | 45 d | 0.311 mM 6-Benzylaminopurine(6-BA) for 10 min | 6-BA treatment inhibits autophagic degradation of Rubisco, thereby alleviating chlorophyll degradation in *Brassica rapa* Subsp. *Chinensis*. | [39] |
| GA | *Brassica rapa* subsp. *chinensis* | 45 d | 0.231 mM GA for 10 min | GA treatment inhibits autophagic degradation of Rubisco, thereby alleviating chlorophyll degradation in *Brassica rapa* Subsp. *Chinensis*. | [39] |
| ABA | *Arabidopsis* | 1week(w) | 50 μM ABA for 3 or 6 h | Free GFP levels are significantly increased in ABA-treated GFP-*ATG8e* seedlings. | [50] |
| ABA | banana | 1 w | 20 μM ABA for 1 d | ABA treatment induces the formation of autophagosomes. | [47] |
| ABA | *Brassica napus* L. | No data available. | ABA for 24h | ABA treatment increases the transcript levels of *BnATGs*. | [48] |
| ABA | *Fragaria vesca* | No data available. | 100μM ABA for 24 h | ABA treatment upregulates FvRD21 expression, promotes its mediated autophagic degradation of viral P6 protein. | [49] |
| SA | *Arabidopsis* | 7 d | 100 μM BTH for 8 h | Many structures possibly representing autophagosomes were detected in root cells after BTH treatment. | [70] |
| SA | *Arabidopsis* | 3-4 w | 100 μM SA or 100 μM MeSA for 12 h | SA or MeSA can induce more autophagosome formation. | [71] |
| SA | *Arabidopsis* | 5 w | 10 μM SA for 5 d | More ATG8-labeled autophagosomes were observed under SA treatment. | [75] |
| SA | *Arabidopsis* | 3 w | 200 μM SA for 4 d under constant dark treatment | Exogenous SA inhibits autophagy via NPR1 to accelerate leaf senescence under carbon starvation stress. | [74] |
| SA | *Solanum tuberosum* | 4 w | 1 mM SA for 12 h | *SA treatment upregulates the expression of StATG3, StATG9, StATG11, StATG13a and StATG8-2.1.* | [73] |
| SA | *Arabidopsis* | No data available. | Leaves were infiltrated with 1 mM SA for 4-24 h | SA treatment increases ATG8-PE protein levels. | [72] |
| ETH | *Ipomoea nil* | Flowers were excised at 20:00 h on the day before flower opening | Flowers were placed into closed clear plastic chambers containing 1 μL L^–1^ ETH. | ETH treatment upregulates *InATG4b* but not *InATG8a* expression and accelerates floral senescence. | [105] |
| ETH | Petunia | 1 d after flower | 0.1、1.0、10.0 μL/L ETH for 4 h | ETH treatment elevates the transcript abundance of multiple *ATGs*. | [106] |
| ETH | *Petunia hybrida* | After flower | 2 μL/L ETH for 4、16 or 24 h | *PhATG8* homolog expression correlates positively with ETH treatment duration. | [108] |
| ETH | *Solanum lycopersicum* | 5 w | 0.1 μΜ ACC and dehydration by withholding water for 7d | After treatment, the number of autophagosomes increased and autophagic activity enhanced, with *ATG8d* and *ATG18h* significantly induced. | [109] |
| ETH | *Glycine max* [L.] Merr. cv. Fukuyutaka | No data available. | 100 μΜ ACC for 12 or 24 h | The expression of *GmATG8i* and *GmERF* was up-regulated after treatment. | [107] |
| ETH | *Citrus sinensis* cv. Newhall | Fruits at the mature green stage | Fruits were immersed in a 1000 mg/ L ethephon solution | Spraying unripe citrus with ethephon accelerates fruit yellowing and enhances autophagy activity. | [112] |
| ETH | *Arabidopsis* | 4 d | Seedlings were incubated in 20μM ACC for 2 h in the dark | Enhanced autophagy activity and increased autophagosome numbers after treatment indicate that ETH itself triggers autophagy. | [104] |
| SL | *Solanum lycopersicum* L. | 5 w | the tomato roots were treated with 3 μM GR24^5DS^，24 h before cold treatment. | Enhanced autophagy activity (more autophagosomes and upregulated expression of *ATGs*) after treatment significantly improved plants cold resistance. | [116] |
| BR | *Arabidopsis* | 7 d | 100 nM BL （brassinolide the most biologically active member of the BR family) for 3 d | BL treatment significantly reduces autophagosome numbers in plants under sugar starvation. | [120] |
| BR | *Arabidopsis* | 7 d | 1 μM brassinazole (BRZ, a BR biosynthesis inhibitor) for 3 d | BRZ treatment increases autophagosome numbers in plants under sufficient sucrose conditions. | [120] |
| BR | *Prunus persicae L.* | 1 year(y) | 1 μM 24-epibrassinolide (EBR) | EBR treatment reduces *PpATG* expression levels and autophagosome numbers. | [121] |
| BR | *Solanum lycopersicum* | 6 w | 500 nM BL for 3-24 h | The transcription levels of *ATGs* slightly increased at 3 h, peaked at 12 h, and most returned to the control level after 24 h. | [122] |
| BR | *Solanum lycopersicum* | 6-week-old tomato | 500 nM BL for 12 h | After treatment, the number of autophagosomes increased by 9.3 times. | [122] |
| BR | *Solanum lycopersicum* | 5-week-old tomato | 200 nM BL | Foliar application of BL increased cold-responsive autophagosomes and significantly induced autophagic activity. | [123] |
| BR | *Vitis vinifera* L. | Two-year-old cuttings of *Vitis vinifera* L. | exogenous EBR pretreatment for 13 days | EBR pretreatment enhances autophagy activity, facilitating the degradation of damaged chloroplasts to alleviate the adverse effects of drought stress. | [124] |
| melatonin | *Malus hupehensis* Rehd | 3-month-old | 100 μM melatonin solution in water applied once every 6 days during the entire two-month experiment | After treatment, the expression of *ATGs* (e.g., *MdATG3*, *MdATG7*) in leaves is reduced, autophagy activity is inhibited, protein and organelle degradation is slowed down, and leaf senescence is significantly delayed. | [146] |
| melatonin | *Solanum lycopersicum* L. cv Ailsa Craig | 8 w | plants were sprayed with 10 mL of 10 μM melatonin 8 hr  prior to the imposition of high temperature treatment | Melatonin pretreatment induces the expression of *ATGs* (e.g., *ATG5*, *ATG8*), promotes autophagosome formation, and alleviates various cell damages caused by high temperature. | [138] |
| melatonin | *Solanum lycopersicum* | when the first inflorescence of tomato plants were fully developed, and the other inflorescences were budded | tomato seedlings were pretreated with 20 µM melatonin for 7 days, followed by 42 °C high temperature for 3 h | Melatonin pretreatment of tomato significantly activates *ATGs* (e.g., *ATG6*, *ATG8c*), promotes autophagosome formation, and mitigates the damage of high temperature to pollen development. | [139] |
| melatonin | cassava | 1-month-old | 100 μM me-  latonin with 5 ‰ SilWet L-77 for 1 and 6 hours | Melatonin induces the expression of multiple *ATGs* and increases the number of autophagosomes. | [145] |
| melatonin | *Pyrus pyrifolia* | Fruits | Fruits were immersed in 100 μM of melatonin containing 0.05% Tween 20 for 6 h. | Melatonin treatment enhances the expression of core ATGs and autophagosome formation in fruits, thereby improving resistance to *Botryosphaeria dothidea*. | [141] |
| melatonin | *Gossypium hirsutum* L. | Seeds | Seeds were surface sterilized and imbibed with 10 μM of melatonin for 24 h in the dark | Melatonin treatment significantly upregulates *ATG8c/8f* expression, increases ATG8-PE protein content, and enhances plant drought tolerance. | [142-144] |
| melatonin | *Arabidopsis* | 7 d | Seedlings were pretreated with 10 μM melatonin，and then transferred to MS medium containing 10 μM methyl viologen for 48 h | Under methyl viologen-induced oxidative stress, melatonin-pretreated *Arabidopsis* exhibits more autophagosomes in roots and higher expression of *ATG8* isoforms. | [137] |

Table S3: Regulation of Autophagy by endogenous phytohormones.

| Phytohormones | Species | Effect | Reference |
| --- | --- | --- | --- |
| Auxin | *Triticum aestivum* L. | Three IAA-related *cis*-acting elements are present in the promoters of *ATGs*. | [28] |
| Auxin | *Oryza sativa* L. ssp. Japonica cv. Zhonghua 11 | The promoters of rice ATGs contain 17 auxin-responsive *cis*-acting elements. | [29] |
| Auxin | *Arabidopsis* | The promoter regions of *ATG8A* and *ATG8H* contain auxin response factor family binding sites. | [30] |
| GA | *Triticum aestivum* L. | Three GA-related *cis*-acting elements are present in the promoters of *ATGs*. | [28] |
| GA | *Oryza sativa* L. ssp. Japonica cv. Zhonghua 11 | The promoters of rice *ATGs* contain 20 GA-responsive *cis*-acting elements. | [29] |
| ABA | *Triticum aestivum* L. | Three ABA-related *cis*-acting elements are present in the promoters of *ATGs*. | [28] |
| ABA | *Oryza sativa* L. ssp. Japonica cv. Zhonghua 11 | The promoters of rice *ATGs* contain 34 ABA-responsive *cis*-acting elements. | [29] |
| ABA | *Arabidopsis* | ABA-dependent signaling induced by mannitol can reduce the sulfation modification of ATG4a, thereby activating the protein and promoting autophagy. | [50] |
| ABA | *Arabidopsis* | ABA activates SnRK2, which phosphorylates RaptorB, a component of the TOR complex, leading to complex dissociation and relief of TOR-mediated autophagy inhibition. | [45] |
| SA | *Arabidopsis* | Autophagy induced during cucumber mosaic virus infection is mediated by endogenous SA. | [76] |
| SA | *Arabidopsis* | SA-dependent autophagy contributes to plant defense against bacterial infection. | [77] |
| SA | *Arabidopsis* | Submergence-induced autophagosome formation is blocked in *sid2* and *npr1-5*, indicating that this process is dependent on the SA-NPR1 signaling pathway. | [78] |
| SA | *Arabidopsis* | The *npr3 npr4* double mutant exhibits more severe premature senescence and reduced autophagosome production compared with wild type, indicating that NPR3 and NPR4 positively regulate plant autophagy synergistically. | [79] |
| SA | *Triticum aestivum* L. | Two SA-responsive *cis*-elements are present in the promoter regions of *ATGs*. | [28] |
| SA | *Oryza sativa* L. ssp. Japonica cv. Zhonghua 11 | The promoters of rice *ATGs* contain 13 SA-responsive *cis*-acting elements, and 5 *ATGs* show significant expression changes after SA treatment. | [29] |
| JA | *Solanum lycopersicum* | Autophagy is inhibited and cell death is reduced in the JA receptor mutant *jai1*, indicating that JA signaling can promote plant autophagy. | [97] |
| JA | *Triticum aestivum* L. | The promoters of ATGs contain two methyl JA-responsive elements. | [28] |
| JA | *Oryza sativa* L. ssp. Japonica cv. Zhonghua 11 | The promoters of rice *ATGs* contain 30 JA-responsive *cis*-acting elements, and 5 *ATGs* show significant expression changes after JA treatment. | [29] |
| JA | *Arabidopsis* | JA promotes petal abscission by inducing autophagy. | [96] |
| ETH | *Petunia hybrida* | Pollination-induced elevation of endogenous ETH content induces the expression of *PhATG8* homologs. | [108] |
| ETH | *Solanum lycopersicum* | ETH induced by drought stress enables ERF5 protein to bind the DRE elements in *ATG8d* and *ATG18h* promoters, activate gene transcription and promote autophagy. | [109] |
| ETH | *Triticum aestivum* L. | The promoters of *ATGs* contain one ETH-related element. | [28] |
| ETH | *Oryza sativa* L. ssp. Japonica cv. Zhonghua 11 | The promoters of rice *ATGs* contain 16 ETH-responsive *cis*-acting elements. | [29] |
| ETH | *Arabidopsis* | The promoters of *ATG8A* and *ATG8H* contain binding elements for transcription factors of the APETALA2/ethylene-responsive element binding proteins family. | [30] |
| ETH | *Solanum lycopersicum* | Under drought stress, ERF5 directly binds to the promoters of *ATG8d* and *ATG18h*, activates their expression and promotes autophagy. | [109] |
| ETH | *Arabidopsis* | Yeast one-hybrid assays showed that transcription factors from the APETALA2/ethylene-responsive element binding proteins family were enriched in the screening for *ATG8a* and *ATG8h*. | [30] |
| ETH | *Arabidopsis* | After submergence treatment, ETH content in roots increased significantly, accompanied by elevated autophagy level and expanded autophagy range from vascular tissues to epidermis. | [111] |
| BR | *Arabidopsis* | BAK1 binds to and phosphorylates ATG18a, resulting in the inhibition of autophagic activity. | [119] |
| BR | *Vitis vinifera* cv. Thompson Seedless | BR-activated VvBZR1 binds to the *VvATG18a* promoter, activates its expression, enhances autophagy and improves disease resistance. | [125] |
| BR | *Solanum lycopersicum* L. | The *ccd7* mutant (deficient in a key enzyme for SL biosynthesis) produces significantly fewer autophagosomes than the wild type under cold stress, exhibiting higher cold sensitivity and weaker cold tolerance. | [116] |
| melatonin | *Solanum lycopersicum* L. cv Ailsa Craig | Overexpression of melatonin synthesis gene *ASMT* induces the expression of *ATGs* (e.g., *ATG5*, *ATG8*), promotes autophagosome formation. | [138] |
| melatonin | *Medicago sativa* L. cv. Biaogan | Heterologous expression of alfalfa melatonin synthesis gene *MsSNAT* in *Arabidopsis* significantly increased melatonin production, enhanced root autophagosome accumulation and *ATGs* expression. | [140] |
| melatonin | cassava | Melatonin synthase overexpression promotes autophagosome accumulation and enhances autophagic activity, while its silencing exerts the opposite effect. | [145] |
